# Mechanisms for the anti-obesity actions of bofutsushosan in high-fat diet-fed obese mice

**DOI:** 10.1186/s13020-017-0129-x

**Published:** 2017-03-27

**Authors:** Shinjiro Kobayashi, Yuki Kawasaki, Tatsuo Takahashi, Hironori Maeno, Masaaki Nomura

**Affiliations:** 1 Department of Clinical Pharmacy, Faculty of Pharmaceutical Sciences, Hokuriku University, Kanazawa, 920-1181 Japan; 2 Center of Clinical Pharmacy Education, Faculty of Pharmaceutical Sciences, Hokuriku University, Kanazawa, 920-1181 Japan

**Keywords:** Bofutsushosan (BTS), High-fat diet-fed obese mice, Adiponectin mRNA, Leptin mRNA, UCP1 mRNA, Insulin resistance, White adipocyte, Brown adipocyte

## Abstract

**Background:**

The Kampo medicine bofutsushosan (BTS; *Pulvis ledebouriellae compositae*; *Fang Feng Tong Sheng San*) has been used as an anti-obesity treatment in overweight patients. In this study, we assessed the underlying physiological changes induced by BTS in obese mice maintained on a high-fat diet.

**Methods:**

Male ICR mice were fed a 60% kcal fat diet for 5 weeks starting at 4 weeks of age and then fed the same diet with administration of water (control) or aqueous BTS extract (1.0–2.0 g/kg) for 25 days. Body weight, wet weight of isolated white adipose tissue, and obesity-related serum parameters (glucose, lipids, leptin, adiponectin) were measured after treatment. The mRNA expression levels of leptin, adiponectin, and UCP1 in the adipose tissues were determined by quantitative real-time polymerase chain reaction after the first 5 days of treatment.

**Results:**

Bofutsushosan (1.5–2.0 g/kg) significantly decreased total body weight and total wet weight of white adipose tissue isolated from subcutaneous (retroperitoneal) and visceral regions (epididymal, mesenteric, and perirenal). At 2.0 g/kg, BTS also decreased total fat mass, visceral fat mass, and ratio of fat mass to body weight as measured by computed tomography, and significantly decreased epididymal adipocyte size after 14 and 25 days’ treatment. Twenty-five days’ treatment lowered serum glucose, insulin, leptin, and triglycerides, and reduced homeostasis model assessment-insulin resistance. Alternatively, 2.0 g/kg BTS significantly increased mRNA levels of adiponectin, leptin, and UCP1 in interscapular brown adipose tissue but not epididymal white adipose tissue after 5 days’ administration.

**Conclusion:**

In the early administration period, BTS increased mRNA expression levels of leptin, adiponectin, and UCP1 in brown adipose tissues. With longer administration, BTS improved insulin resistance, and subsequently reduced serum levels of leptin and triglyceride in parallel with decreased visceral white adipose tissue volume and adipocyte size.

**Electronic supplementary material:**

The online version of this article (doi:10.1186/s13020-017-0129-x) contains supplementary material, which is available to authorized users.

## Background

Obesity rates are increasing worldwide [1], resulting in progressively greater incidences of associated health problems such as type 2 diabetes mellitus, ischemic heart disease, stroke, and cancer. Obesity results from an imbalance between food intake and energy consumption (basal metabolism and volitional energy expenditure). Excessive caloric intake due to the ample availability of energy-dense meals is thought to be the main contributor to global obesity [2].

Foods that are rich in fats also increase body weight induce diabetes in mice and rats [3]. Indeed, many studies have characterized the pathological responses of animals to high-fat diets [4, 5]. Although the pathogenesis of obesity is complex; the key factor is long-term deregulation of energy balance due to increased energy intake and/or reduced energy expenditure. Mammals have evolved chemical energy-storing white adipose tissue and energy-dissipating brown adipose tissue [6, 7] to regulate energy balance. White adipose tissue enables humans to survive for longer periods between meals, storing energy mainly as triglycerides, and releasing fatty acids during fasting. White adipocytes contain a large single, spherical lipid vacuole, and a peripherally located nucleus but few mitochondria. Fat storage is regulated by complex endocrine signaling between white adipose tissue and the central nervous system (CNS), mediated by hormones such as leptin and adiponectin. Several groups have demonstrated that administration of adiponectin increases fatty acid oxidation in muscle and decreases hepatic glucose production, resulting in amelioration of insulin resistance and improved glucose metabolism in diabetic mice. Conversely, decreased adiponectin and increased leptin in serum contribute to the development of metabolic complications in obesity, particularly diabetes and insulin resistance [8–13].

In addition to lifestyle interventions, obese individuals can be treated by pharmacological therapy. The complex Kampo herbal formulation bofutsushosan (BTS; *Pulvis ledebouriellae compositae*; *Fang Feng Tong Sheng San*), consisting of 18 crude components, has been used as an anti-obesity drug in overweight patients, where it reduces hypertension, insulin resistance, and subcutaneous abdominal fat thickness. In addition, BTS has been reported to exert anti-obesity effects in various obese and diabetic mouse models [14–16]. We have investigated the anti-diabetic actions of BTS in the streptozotocin (STZ) diabetic mouse and found efficacy similar to that of the anti-diabetic drug [17, 18]. One BTS component, Gardenia, improved insulin resistance and repaired insulin signaling via upregulation of P-Akt, glucose transporter 4, and glucose uptake in skeletal muscle of STZ-diabetic mice [19]. However, anti-obesity actions of BTS were not investigated in STZ-diabetic mice.

Uncoupling protein (UCP) 1 is specifically expressed in the mitochondrial inner membrane of brown adipocytes, where it generates heat by uncoupling oxidative phosphorylation. The induction of thermogenesis is controlled by the cold–warm centers in the CNS through β_3_ adrenergic signaling. The expression of UCP1 therefore regulates thermogenesis, energy expenditure, and oxidative stress, processes associated with the pathogenesis of obesity [20, 21]. Upregulation of UCP1 by genetic manipulations or pharmacological agents can reduce obesity and improve insulin sensitivity [22, 23]. In the present study, we investigated the effects of BTS on the mRNA expression levels of UCP1, adiponectin, and leptin in adipose tissue, as well as insulin resistance, serum triglycerides, white adipocyte size, and white adipose tissue weight in high-fat diet-fed obese model mice.

## Methods

### Animals

**Fig. 1:**
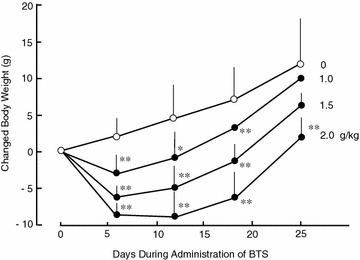
Effects of BTS on body weight in high-fat diet-fed obese mice. ICR mice (4 weeks of age) were provided a high-fat diet for 5 weeks ad libitum. Doses (0, 1.0, 1.5, and 2.0 g/kg) of BTS were administered orally to obese mice at 9 weeks of age for 25 days under continued high-fat diet. Body weights were measured on days 0, 6, 12, 18, and 25 during administration. Body weight changes were calculated from day 0 before administration of BTS to day 25. Values are expressed as mean ± SD (n = 6–8). Differences between group means were evaluated by Tukey’s or Scheffe’s tests. *P < 0.05, **P < 0.01: significantly different from control water group without BTS

**Fig. 2:**
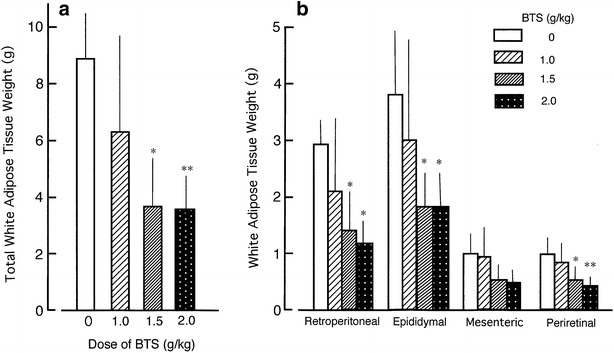
Effects of BTS on total white adipose tissue wet weight (**a**) and individual wet weights of retroperitoneal, epididymal, mesenteric, and perirenal white adipose tissues (**b**) in obese mice orally administered various doses (0, 1.0, 1.5, and 2.0 g/kg) of BTS for 25 days under a high-fat diet. White adipose tissues were isolated from obese mice and wet weights measured. Values are expressed as mean ± SD (n = 5–7). Differences between group means were evaluated by Tukey’s or Scheffe’s tests. *P < 0.05, **P < 0.01: significantly different from control water group without BTS

**Fig. 3:**
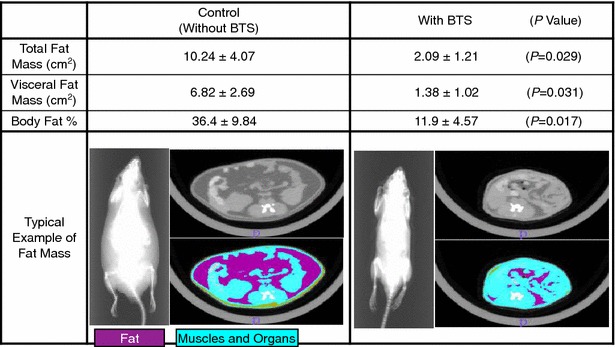
Effects of BTS (2.0 g/kg) on total fat mass, visceral fat mass, and body fat percentage in obese mice measured by computed tomography. Shown is a typical example of fat mass. *Purple areas* are fat masses, and *blue areas* are skeletal muscle and organ masses. Body fat percentage was calculated as [area of fat mass]/[(area of fat mass) + (area of muscle and organ masses)] × 100. Values expressed as mean ± SD (n = 3). *P* values in parenthesis were calculated by Student’s *t* test. *P* < 0.05, BTS group is significantly different from control group without BTS

**Fig. 4:**
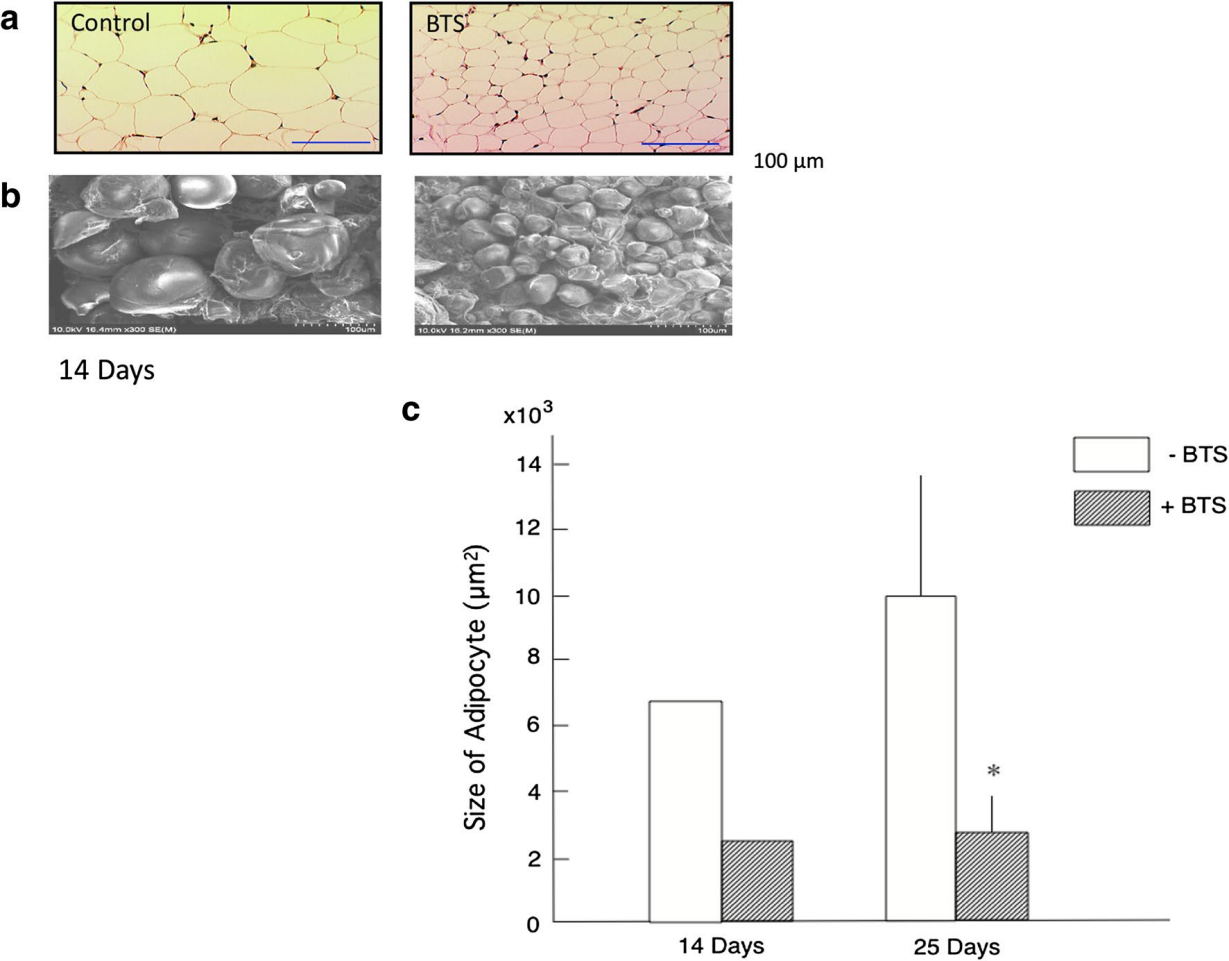
Effects of BTS (2.0 g/kg) on visceral epididymal adipocyte size under optical microscopy (**a**) and scanning electron microscope at ×300 magnification (**b**) after 14 days’ administration and epididymal adipocyte size under optical microscopy after 14 and 25 days (**c**). Values are expressed as mean ± SD (n = 1 and 3, respectively). Difference between group means was evaluated by Student’s *t* test. *P < 0.05: significantly different from control water group without BTS

**Fig. 5:**
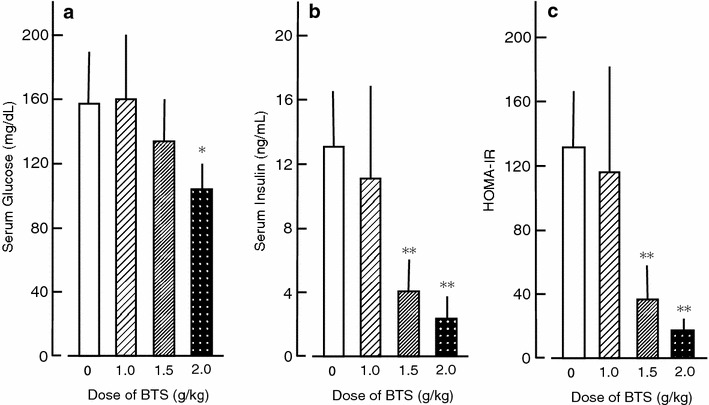
Effects of BTS on serum glucose (**a**), serum insulin (**b**) and HOMA-IR value (**c**) in obese mice. HOMA-IR value was calculated as [serum glucose (mg/dL) × serum insulin (μU/mL)]/405. Doses of BTS (0, 1.0, 1.5, and 2.0 g/kg) were administered orally to obese mice (9 weeks of age) for 25 days under a high-fat diet. Values are expressed as mean ± SD (n = 6). Differences between group means were evaluated by Tukey’s or Scheffe’s tests. *P < 0.05, **P < 0.01: significantly different from control water group without BTS

**Fig. 6:**
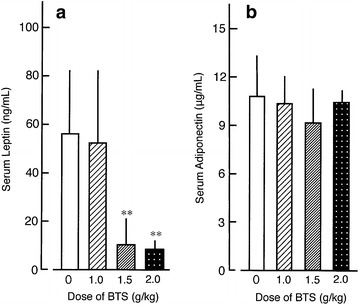
Effects of BTS on serum leptin (**a**) and serum adiponectin (**b**) in obese mice. Doses of BTS (0, 1.0, 1.5, and 2.0 g/kg) were administered orally to obese mice (9 weeks of age) for 25 days under a high-fat diet. Values expressed as mean ± SD (n = 6–7). Differences between group means were evaluated by Tukey’s or Scheffe’s tests. **P < 0.01: significantly different from control water group

**Fig. 7:**
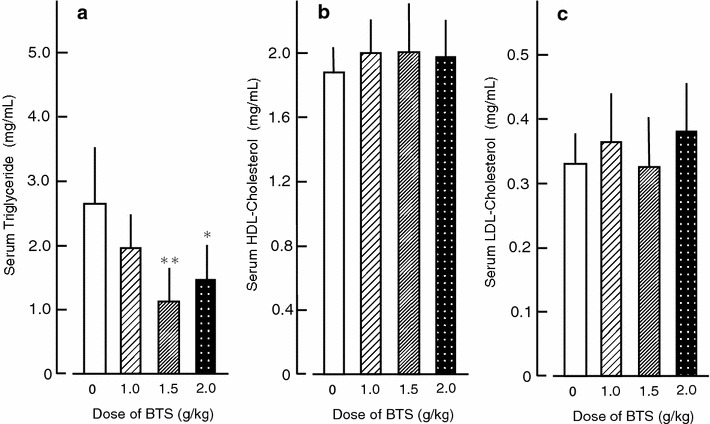
Effects of BTS on serum triglyceride (**a**), serum HDL-cholesterol (**b**), and serum LDL-cholesterol (**c**) in obese mice. Doses of BTS (0, 1.0, 1.5, and 2.0 g/kg) were administered orally to obese mice (9 weeks of age) for 25 days under a high-fat diet. Values are expressed as mean ± SD (n = 6–7). Differences between group means were evaluated by Tukey’s test. *P < 0.05, **P < 0.01: significantly different from control water group without BTS

Male ICR mice (8 weeks of age) fed a 60% kcal fat diet (D12492, Research Diets Inc, New Brunswick, USA) for 4 weeks from 4 weeks of age were purchased from Japan SLC (Shizuoka, Japan). Mice were acclimated for 1 week in a conventional mouse breeding room at Hokuriku University with water ad libitum and kept at 25–26 °C with lights on from 7 a.m. to 7 p.m. Mice were then randomly divided into four groups, one control group and three BTS dosage groups, with eight mice per group. The animal studies were performed following the ARRIVE guideline (Additional file 1). The Ethics Review Committee for Animal Experimentation of Hokuriku University, Kanazawa, Japan approved the experimental protocol (Additional file 1). The studies were also provided information described in Minimum Standards of Reporting Checklist (Additional file 2) and summary of all datasets in Figs. 1, 2, 3, 4, 5, 6 and 7, and Table 2 (Additional file 3).

### Preparation and administration of drugs

Bofutsushosan was prepared from 18 crude components: Glycyrrhiza, Rhubarb, Gardenia, Ephedra, Mirabilite, Forsythia, Ginger, Scutellaria, Schizonepeta, Platycoden, Mentha, Peony, Atractylodes, Cnidium, Glypsum, Saposhnikovia, Angelica, and Talcum (Table 1) [14–16]. Prepared extract of BTS was a free gift from Kobayashi Pharmaceutical Co Ltd. (Ibaraki, Osaka, Japan). A mixture of cut crude components (Table 1) was extracted in 10 volumes of distilled water for 30 min at >90 °C. The extracted solution was filtered, concentrated by vacuum, and spray-dried. The yield of dried BTS extract was 18.5% (w/w) and contained 0.80% geniposide, 2.77% baicalin, 0.1% sennoside A, 0.14% total alkaloids, 0.71% glycyrrhizic acid, and 0.26% paeoniflorin. For experiments, BTS doses of 1.0, 1.5, and 2.0 g/kg body weight were selected because lower doses (0.25 and 0.5 g/kg) did not affect the body weight of obese model mice. Solutions containing 20, 30, and 40 mg/mL were prepared for the 1.0, 1.5, and 2.0 g/kg dose groups, respectively, as the obese mice (ca. 60 g) drank approximately 3 mL of water/day with or without BTS. These solutions of BTS extracts were totally soluble in the water. The stability of BTS extracts in the water is not confirmed but the extracted solutions were prepared every day for 25 days in such a way as to any other traditional Chinese medicine did. Solutions were administered orally to the 9-week old obese mice for 25 days under free intake of the high-fat diet while control group mice received equivolume water under the same high-fat diet. The volume of BTS solution consumed was checked daily and an insufficient volume was directly administered by syringe up to 3 mL/day. Body weights of BTS-treated and control mice were measured on day 0, 6, 12, 18, and 25 of administration.

**Table 1:** Components of bofutsushosan (BTS)

Components	Component rate (%)
Glycyrrhizae Radix (Glycyrrhiza)	7.39^a^
Rhei Rhizoma (Rhubarb)	5.55
Gardeniae Fructus (Gardenia)	4.41
Ephedrae Herba (Ephedra)	4.41
Natrium Sulfuricum (Mirabilite)	5.55
Forsythiae Fructus (Forsythia)	4.41
Zingiberis Rhizoma (Ginger)	1.13
Scutellariae Radix (Scutellaria)	7.39
Schizonepetae Spica (Schizonepeta)	4.41
Platycodi Radix (Platycoden)	7.39
Menthae Folium (Mentha)	4.41
Paeonia Radix (Peony)	4.41
Atractyloids Lanceae Rhizoma (Atractylodes)	7.39
Cnidii Rhizoma (Cnidium)	4.41
Gypsum Fibrosum (Gypsum)	7.39
Saposhnikoviae Radix (Saposhnikovia)	4.41
Angelicae Radix (Angelica)	4.41
Talcum Crystallinum (Talcum)	11.1

^a^Each value (%) is the component rate of BTS

### Measurement of serum glucose, insulin, leptin, adiponectin, triglyceride, and cholesterol

Blood was collected from the neck vein plexus of anesthetized mice after administration of BTS solution or water for 25 days. Blood samples were centrifuged at 2000*g* for 5 min at 25 °C and the supernatant was used as the serum sample. Serum glucose concentration was measured by the glucose oxidase method using a serum glucose monitor (Medisafe Mini, Termo, Tokyo, Japan). Serum insulin was measured using a mouse enzyme linked immunosorbent assay (ELISA) kit for insulin (Morinaga, Yokohama, Japan). The homeostasis model assessment-insulin resistance (HOMA-IR) value was calculated as [serum glucose (mg/dL) × serum insulin (μU/mL)]/405 [24]. Serum leptin and adiponectin were measured with mouse Leptin and Adiponectin/Acrp30 Quantikine ELISA kits (R&B Systems, Minneapolis, USA). Serum triglyceride was measured using the Wako Triglyceride E Test (Wako, Osaka). High-density lipoprotein (HDL) cholesterol and low-density lipoprotein (LDL) cholesterol were measured using HDL and LDL/VLDL-cholesterol Quantification Kits (Bio Vision, Milpitas, USA).

### Isolation of white and brown adipose tissue

After treatment, mice were euthanized by cervical dislocation, and white adipose tissues isolated from the retroperitoneal subcutaneous region and the epididymal, mesenteric, and perirenal viscera. Wet weights of these isolated adipose tissue samples were measured. In some experiments, epididymal white adipose tissue and interscapular brown adipose tissue were isolated from euthanized obese mice administered BTS (2.0 g/kg) or water for 5 days and stored in ISOGEN reagent (Nippon Gene, Tokyo) for gene expression analysis (below).

### Measurement of total, subcutaneous, and visceral fat masses and percent body fat

Total fat, subcutaneous fat, visceral fat, and muscle/organ masses were measured using a computed tomography system (Aloka, Tokyo) in euthanized obese mice following 25 days’ treatment. Percent body fat was calculated using the following formula: [area of fat mass]/[(area of fat mass) + (area of muscle and organ masses)] × 100.

### Measurement of adipocyte size in epididymal adipose tissue

Small pieces of epididymal adipose tissue were isolated from euthanized obese mice after 14 and 25 days’ treatment with BTS or water, fixed with 10% formalin, embedded in paraffin, sectioned, and stained with hematoxylin and eosin. To estimate the size of adipocytes, cross-sectional areas of six randomly selected adipocytes per section were measured at magnification using ImageJ software (National Institutes of Health, Bethesda, USA).

### Scanning electron microscopy

Epididymal adipose tissues from euthanized obese mice treated with BTS or water for 14 days were fixed with 2% glutaraldehyde in HEPES buffer, post-fixed in 2% osmium tetroxide, incubated in 50, 70, 90, 95, and 100% ethanol, and dehydrated in butanol. After lyophilization and sectioning, tissues were examined by scanning electron microscopy at 300× magnification.

### Quantitative real-time PCR for mRNA analysis

The mRNA expression levels of UCP1, UCP2, leptin, adiponectin, and β-actin (as an internal control) in interscapular brown and epididymal white adipose tissues were determined by quantitative real-time polymerase chain reaction (qRT-PCR). Total RNA was extracted using ISOGEN reagent according to the manufacture’s instruction (Wako). First-strand cDNAs were synthesized using Moloney Murine Leukemia Virus (M-MLV) reverse transcriptase (Invitrogen, Carlsbad, CA). Reverse transcription (RT) reactions were carried out in 75 mM KCl, 50 mM Tris–HCl (pH 8.3), 3 mM MgCl_2_, 10 mM dithiothreitol (DTT), 0.5 mM each of dATP, dCTP, dGTP, and dTTP, 40 units of recombinant ribonuclease inhibitor (RNaseOUT™, Invitrogen), 0.5 μg of Oligo(dT)_12–18_ primer (Invitrogen), total RNA, and 200 units of the M-MLV reverse transcriptase in a final volume of 20 μL at 37 °C for 50 min. Real-time PCR was performed in a final volume of 25 μL containing 0.5 μM forward and reverse primers, 12.5 μL GeneAce SYBR qPCR Mix Low Rox (Nippon Gene) and 1 μL of prepared cDNA template using an ABI 7500 Real-Time PCR System (Applied Biosystems, Foster City, CA). The primers were designed using Primer Express software (Applied Biosystems) and had the following sequences: for UCP1, forward 5′-ACAGAAGGATTGCCGAAACTGT-3′ and reverse 5′-GTCGTAGAGGCCAATCCTGAGT-3′ (69 bp product); for UCP2, forward 5′-CTCCCTTGCCACTTCACTTCTG-3′ and reverse 5′-CATGTATCTCGTCTTGACCACATCA -3′ (66 bp product); for leptin, forward 5′-TCAAGCAGTGCCTATCCAGAAA-3′ and reverse 5′-GGTGAAGCCCAGGAATGAAG-3′ (146 bp product); for adiponectin, forward 5′-TGTATCGCTCAGCGTTCAGTGT-3′ and reverse 5′-TCCCGGAATGTTGCAGTAGAA-3′ (140 bp product); for β-actin, forward 5′-AGGGAAATCGTGCGTGACAT-3′, and reverse 5′-GAACCGCTCGTTGCCAATAG-3′ (131 bp product). Target gene expression was calculated relative to β-actin expression.

### Statistical analyses

All values are expressed as mean ± SD. Differences between group means were evaluated by one-way analysis of variance followed by Tukey’s or Scheffe’s multiple range tests using Mac Toukei-Kaiseki Ver.2.0 (Esumi, Tokyo). When variance within each group is similar or different between groups, Tukey’s test or Scheffe’s test was used respectively. In some experiments, differences between two groups were analyzed by Student’s *t* test. A value of P < 0.05 was considered statistically significant from the control water group without BTS.

## Results

### Effects of BTS on body and adipose tissue weights in high-fat diet-fed obese mice

The high-fat diet (D12492) significantly increased body and adipose white tissue weights of ICR mice compared to normal diet (D12450) (data not shown). Changed body weight in vertical axis of Fig. 1 was shown as difference from body weight of same mice at day 0 without administration of BTS. Body weight change (day 6 to day 25) was significantly reduced in the obese mouse group administered BTS (1.0–2.0 g/kg) (Fig. 1). At day 25, Body weight was significantly reduced in the obese mouse group administered 2.0 g/kg BTS compared to the water-administered control group [45.9 ± 3.8 g (n = 7) vs. 59.0 ± 9.5 g (n = 6), P < 0.05] but not in groups receiving 1.0 or 1.5 g/kg BTS [55.9 ± 8.5 g (n = 8) and 48.0 ± 4.6 g (n = 6)]. In addition, the wet weights of white adipose tissues isolated from the subcutaneous retroperitoneal area and epididymal, and perirenal viscera were significantly lower in the 1.5 and 2.0 g/kg BTS groups compared to control obese mice (Fig. 2). In contrast, BTS without Gardenia (2.0 g/kg) did not influence body weight or total white adipose tissue wet weight (data not shown). Figure 3 shows typical computed tomography results from mice treated with BTS for 25 days (purple areas show fat masses and blue areas skeletal muscle and organ masses). BTS (2.0 g/kg) significantly reduced total fat mass area, visceral fat mass area, and percent body fat compared to water controls. These results suggest that BTS decreases the body weight of obese mice, at least in part, by reducing white adipose tissue weight.

### Effects of BTS on adipocyte size

Administration of 2.0 g/kg BTS for 14 days appeared to reduce epididymal adipocyte size compared to controls as revealed by both light microscopy (Fig. 4a) and scanning electron microscopy (Fig. 4b). Quantitative analysis indicated that BTS significantly reduced average epididymal adipocyte area after 25 days’ treatment (Fig. 4c). These results demonstrate that BTS can inhibit hypertrophy of visceral adipocytes (a sign of lipid storage) in obese mice.

### Effects of BTS on the mRNA expression levels of leptin, adiponectin, UCP1, and UCP2 in brown and white adipose tissue

Expression levels of leptin, adiponectin, UCP1, and UCP2 were compared between interscapular brown adipose tissue and epididymal white adipose tissue in control obese mice. Expression levels of leptin, adiponectin, and UCP2 mRNAs were similar in brown and white adipose tissue. However, expression of UCP1 mRNA was 1500-fold greater in brown adipose tissue than white adipose tissue (Table 2). Administration of BTS (2.0 g/kg) to obese mice for 5 days significantly increased mRNA expression levels of leptin, adiponectin, and UCP1 in brown adipose tissue (Table 2) compared to water-treated controls, with greater facilitation of leptin and adiponectin compared to UCP1. In contrast, BTS did not affect mRNA expression levels of these factors in white adipose tissue (Table 2). However, there was no changes in the wet weights of brown and white adipose tissues in obese mice during the 5 day administration of BTS (unpublished observations).

**Table 2:** Effects of BTS on mRNA expression of leptin, adiponectin, UCP1, and UCP2 in interscapular brown adipose tissue and epididymal white adipose tissues after 5 days’ administration

	−BTS	+BTS	*P* value
Interscapular brown adipose tissue			
Leptin	0.0264 ± 0.0080	0.1182 ± 0.0612	0.0004
Adiponectin	0.7679 ± 0.2436	2.5412 ± 2.1369	0.0250
UCP1	2.2519 ± 0.9376	3.3746 ± 0.6704	0.0100
UCP2	0.0717 ± 0.0210	0.0833 ± 0.0430	0.4781
Epididymal white adipose tissue			
Leptin	0.0512 ± 0.0243	0.0390 ± 0.0203	0.3756
Adiponectin	0.6115 ± 0.5319	0.9395 ± 0.4682	0.1839
UCP1	0.0015 ± 0.0008	0.0020 ± 0.0014	0.5207
UCP2	0.1560 ± 0.0252	0.1777 ± 0.0930	0.5347

Relative quantification of mRNA expression by real-time PCR was calculated relative to β-actin mRNA. Values are expressed as mean ± SD (n = 4–9). *P* values were calculated by Student’s *t* test. *P* < 0.05, BTS group is considered significantly different from control group without BTS

### Effects of BTS on serum glucose, insulin, leptin, and adiponectin

Serum levels of glucose and insulin in ICR mice fed a normal-fat diet (D12450) were 73.3 ± 33.1 mg/dL and 5.59 ± 2.10 ng/mL, respectively, for a HOMA-IR value of 24.6 ± 11.3. Serum glucose, insulin, and HOMA-IR were all significantly elevated in control obese mice, increases partially reversed in mice receiving BTS (1.0–2.0 g/kg) for 25 days (Fig. 5). Similarly, BTS reduced serum leptin in obese mice (Fig. 6), but did not alter serum adiponectin.

### Effects of BTS on serum triglyceride, HDL-cholesterol, and LDL-cholesterol

BTS (1.5–2.0 g/kg) significantly lowered serum triglyceride levels in obese mice but had no effect on serum HDL-cholesterol or LDL-cholesterol (Fig. 7).

## Discussion

Expansion of visceral adipose tissue in obese individuals contributes to the development of obesity-related disorders such as diabetes mellitus, hyperlipidemia, hypertension, and metabolic syndrome [1, 2]. It is thus critical to suppress visceral adipose tissue growth to prevent these disorders. In the present study, we demonstrate time- and dose-dependent benefits of BTS on visceral adipose tissue weight, obesity-related serum indices, and endocrine signals in high-fat diet-fed obese mice. Consumption of BTS (1.0–2.0 g/kg/day) decreased body weight (Fig. 1), and this reduction was significant in the 2.0 g/kg/day BTS dose group after 25 days’ administration even without diet modification. Daily doses of 1.5 and 2.0 g/kg also significantly reduced accumulation of visceral and subcutaneous adipose tissue after 25 days’ treatment as determined by direct weighing (Fig. 2) and CT examination (Fig. 3) compared to control obese mice. Histological study demonstrated that BTS significantly reduced the average size of epididymal white adipocytes (Fig. 4) compared to controls, suggesting that suppression of visceral white adipocyte hypertrophy accounts for the reductions in tissue and body weight. In contrast to obese mice, BTS (3.0 g/kg/day) had no effect on the body weight and no toxic effect in age-matched ICR normal mice, although some mice have a tendency of increased loose feces (unpublished observation). In addition, BTS without Gardenia (2.0 g/kg, negative control) did not influence body or adipose tissue weight (unpublished observation). Thus, BTS specifically reduced whole-body weight by suppressing the increase in white adipose tissue weight resulting from a high-fat diet. This result parallels observations in obese humans, although the chosen doses of BTS were greater than the human clinical dose (0.095 g/kg) [25].

The mRNA expression level of UCP1 in interscapular brown adipose tissue was approximately 1500-fold greater than that in epididymal white adipose tissue (Table 2), suggesting that the physiological functions of UCP1, such as thermogenesis, predominate in brown adipose tissue of obese mice. UCP1 gene expression has been reported to increase in response to changes in temperature and dietary fat as well as in response to non-esterified fatty acids, adrenergic β_3_-agonists, and high intracellular cyclic AMP [26]. Upregulation of UCP1 by genetic manipulations or pharmacological agents can reduce obesity and improve insulin sensitivity [22, 23]. Administration of BTS for only 5 days upregulated UCP1 mRNA expression 1.5-fold compared to control obese mice, but did not affect the expression level of UCP2 mRNA in either brown or white adipose tissue (Table 2). These results suggest that BTS may increase energy expenditure by promoting UCP1-mediated processes in brown adipose tissues during the early stage of BTS administration. Despite this increase in UCP-1 mRNA expression, there was no changes in the wet weights of brown and white adipose tissues in obese mice during this early period (unpublished observations). These results suggest that BTS may decrease the size and weight of visceral adipose tissue deposits via upregulation of UCP1 mRNA expression in brown adipose tissue of the early period. However, we did not confirm the gene and protein expression changes from the early period to the middle or the end period of the experiment in the present study. Akagiri et al. [15] reported that BTS did not affect UCP1 mRNA expression in brown adipose tissue but increased expression in white adipose tissue during 4 weeks’ administration of BTS to male KK/Ta mice fed a high-fat diet. This suggests that these tissue-specific responses may depend on the experimental model (e.g., obese or diabetic model) and duration of BTS administration.

Hyperphagia and elevated levels of both insulin and leptin are common features of obesity [11–13]. Obesity is associated with resistance to the biological effects of both insulin and the satiety hormone leptin. Serum levels of insulin and leptin were increased in our obese mice, consistent with previous studies [27]. Further, obese mice in this study showed elevated HOMA-IR, indicating insulin resistance. Leptin is a potent inhibitor of feeding and is expected to decrease insulin levels via inhibition of insulin release [28]. In the present study, BTS improved insulin resistance and reduced serum leptin (Figs. 5, 6). In addition, BTS increased leptin mRNA in brown adipose tissue but not in visceral white adipose tissue during the early phase of administration (Table 2). These results suggest that leptin may reduce body weight through direct effects on both brown and white adipose tissue. In brown adipose tissue, increased leptin signaling may upregulate UCP1, leading to enhanced energy utilization and fat oxidation, while increased serum leptin may reduce visceral white adipose tissue by enhancing triglyceride hydrolysis and lipid oxidation and by suppressing lipogenesis and adipocyte proliferation [29].

Adiponectin has been reported to be produced predominantly by normal white adipocytes but not obesity-associated hypertrophic white adipocytes [30]. The present data showed that BTS increased adiponectin mRNA levels in brown adipose tissue during the early phase of administration (Table 2). Alternatively, BTS did not affect adiponectin mRNA levels in white adipose tissue during early administration (Table 2) and did not influence serum adiponectin during longer administration (Fig. 6). Adiponectin has been reported to inhibit UCP1 gene expression by suppressing β_3_-adrenergic receptor expression in brown adipocytes [31, 32]. It is therefore more likely that BTS upregulates UCP1 in obese mice through enhanced leptin mRNA expression than through enhanced adiponectin mRNA expression in brown adipose tissue.

The present study demonstrates that BTS has multiple anti-obesity effects in obese mice. Bofutsushosan reduced serum triglyceride in obese mice (Fig. 7), in accord with results showing that BTS also decreases liver triglyceride levels in obese mice (data not shown). However, BTS did not reduce serum or liver levels of HDL-cholesterol and LDL-cholesterol (Fig. 7 and unpublished observations). Thus, BTS may contain no cholesterol-lower bioactivity or else the levels of this bioactivity were insufficient in the sample used. Indeed, it is not clear which components of BTS mediate these specific anti-obesity action. BTS is composed with 18 crude components (Table 1), and the BTS extract administered contained 0.80% geniposide from Gardenia, 2.77% baicalin from Scutellaria, 0.1% sennoside A from Rhubarb, 0.14% total alkaloids, 0.71% glycyrrhizic acid from Glycyrrhiza, and 0.26% paeoniflorin from Peony. It has been reported that geniposide is a novel agonist for the glucagon-like peptide-1 (GLP-1) receptor [33]. Geniposide as well as GLP-1 may stimulate brown adipose tissue thermogenesis through hypothalamic adenosine monophosphate-activated protein kinase (AMPK) [34]. Thus, this geniposide component may be responsible for enhanced lipolysis of serum triglycerides in obese mice. This notion is supported by findings that AMPK activation induces lipoprotein lipase (LPL) gene expression through PPARγ1 mRNA synthesis in skeletal muscle [35] and that geniposide may activate hormone-sensitive lipase through a noradrenaline-associated cyclic AMP-dependent mechanism [36]. GLP-1 agonist such as geniposide may also release noradrenaline from the CNS to induce UCP1 expression in brown adipose tissue through noradrenaline-associated cyclic AMP-dependent mechanisms [36]. Ephedrine, an alkaloid in Ephedra, acts on sympathetic nerve terminals and promotes noradrenaline release. Noradrenaline induces thermogenesis in brown adipose tissue via β_3_-adrenergic receptor and lipolysis in white adipose tissue through increased cyclic AMP synthesis. Glycyrrhiza, Forsythia, and Schizonepeta have potent inhibitory effects on phosphodiesterase, resulting in increased intracellular cyclic AMP [14, 25], suggesting that these compound as well as Gardenia act synergistically to increase cyclic AMP signaling and downstream upregulation of UCP1 expression in brown adipose tissue. Emodin, one of the active anthraquinone derivatives in Rhubarb, inhibits production of TNF-α and tyrosine kinase activity [37]. Rheinanthrone, a metabolite of Rhubarb-derived sennoside A, activates macrophages in the colon and increases the secretion of prostaglandin E_2_ [38]. Further studies are warranted to assess the effects of these BTS components on obese-related parameters in mice.

## Conclusions

BTS increased mRNA expression levels of leptin, adiponectin, and UCP1 in brown adipose tissue and improved insulin resistance in obese mice fed a high-fat diet. BTS subsequently reduced the serum levels of leptin and triglyceride with parallel decreases in the size of visceral adipocytes and visceral adipose tissue weight.

## Additional files

**Additional file 1.** The ARRIVE Guidelines Checklist.

**Additional file 2.** Minimum standards of reporting checklist.

**Additional file 3.** Summary of all datasets in Figs. 1, 2, 3, 4, 5, 6 and 7, and Table 2.

## Abbreviations

AMPK: adenosine monophosphate-activated protein kinase
BAT: brown adipose tissues
BTS: bofutsushosan
DTT: dithiothreitol
ELISA: enzyme linked immuno solvent assay
GLP-1: glucagon-like peptide-1
HDL: high density lipoprotein
HEPES: 4-(2-hydroxyethyl)-1-piperazineethanesulfonic acid
HOMA-IR: homeostasis model assessment-insulin resistance
LDL: low density lipoprotein
LPL: lipoprotein lipase
M-MLV: moloney murine leukemia virus
P-Akt: phosphorylated Akt
PCR: polymerase chain reaction
PPAR: peroxisome proliferator-activated receptor
RNaseOUT™: recombinant ribonuclease inhibitor
RT: reverse transcription
SD: standard deviation
STZ: streptozotocin
UCP: uncoupling protein
VLDL: very low density lipoprotein
WAT: white adipose tissues
