# Supplementary material for: Mechanisms for the anti-obesity actions of bofutsushosan in high-fat diet-fed obese mice
Source: Chin Med. 2017 Mar 27;12:8. doi: 10.1186/s13020-017-0129-x (PMC5369197; doi:10.1186/s13020-017-0129-x)
Supplement: Supplementary file 1 — Additional file 1. The ARRIVE Guidelines Checklist. [file 13020_2017_129_MOESM1_ESM.pdf]

ARRIVE

The ARRIVE Guidelines Checklist

Animal Research: Reporting In Vivo Experiments

Carol Kilkenny[1], William J Browne[2], Innes C Cuthill[3], Michael Emerson[4] and Douglas G Altman[5]

[1]The National Centre for the Replacement, Refinement and Reduction of Animals in Research, London, UK, [2]School of Veterinary Science, University of Bristol, Bristol, UK, [3]School of Biological Sciences, University of Bristol, Bristol, UK, [4]National Heart and Lung Institute, Imperial College London, UK, [5]Centre for Statistics in Medicine, University of Oxford, Oxford, UK.

	ITEM	RECOMMENDATION	Section/ Paragraph
Title	1	Provide as accurate and concise a description of the content of the article as possible.	Yes we did.
Abstract	2	Provide an accurate summary of the background, research objectives, including details of the species or strain of animal used, key methods, principal findings and conclusions of the study.	Yes we did.
**INTRODUCTION**			
Background	3	a. Include sufficient scientific background (including relevant references to previous work) to understand the motivation and context for the study, and explain the experimental approach and rationale.<br>b. Explain how and why the animal species and model being used can address the scientific objectives and, where appropriate, the study's relevance to human biology.	Yes we did.
Objectives	4	Clearly describe the primary and any secondary objectives of the study, or specific hypotheses being tested.	Yes we did.
**METHODS**			
Ethical statement	5	Indicate the nature of the ethical review permissions, relevant licences (e.g. Animal [Scientific Procedures] Act 1986), and national or institutional guidelines for the care and use of animals, that cover the research.	This research is covered by the ethics review committee for animal experimen tation of Hokuriku University, Kanazawa, Japan.
Study design	6	For each experiment, give brief details of the study design including:<br>a. The number of experimental and control groups.<br>b. Any steps taken to minimise the effects of subjective bias when allocating animals to treatment (e.g. randomisation procedure) and when assessing results (e.g. if done, describe who was blinded and when).<br>c. The experimental unit (e.g. a single animal, group or cage of animals).<br>A time-line diagram or flow chart can be useful to illustrate how complex study designs were carried out.	a. Same number of animals in each group.<br>b. We carried out randomisation procedure but not blinded test.<br>c. Group of animals.
Experimental procedures	7	For each experiment and each experimental group, including controls, provide precise details of all procedures carried out. For example:<br>a. How (e.g. drug formulation and dose, site and route of administration, anaesthesia and analgesia used [including monitoring], surgical procedure, method of euthanasia). Provide details of any specialist equipment used, including supplier(s).<br>b. When (e.g. time of day).<br>c. Where (e.g. home cage, laboratory, water maze).<br>d. Why (e.g. rationale for choice of specific anaesthetic, route of administration, drug dose used).	a. Water solutions of drug (0, 20,30 and 40 mg/mL), Oral administra tion, Blood collec tion after ether anaesthesia, Isolation of tissues and taking various pictures after euthanized by cervical dislocation<br>b. Administration of drug freely.<br>c. Home cage and laboratory<br>d. Avoidance of excitation by inhalation of ether.

Experimental animals	8	a. Provide details of the animals used, including species, strain, sex, developmental stage (e.g. mean or median age plus age range) and weight (e.g. mean or median weight plus weight range).<br>b. Provide further relevant information such as the source of animals, international strain nomenclature, genetic modification status (e.g. knock-out or transgenic), genotype, health/immune status, drug or test naïve, previous procedures, etc.	a, b. Male mice (4 weeks of age) of ICR strain which were given 60 kcal% fat diet for 5 weeks, Administration of drug, Measurement of changed body weight.

Housing and husbandry	9	Provide details of:<br>a. Housing (type of facility e.g. specific pathogen free [SPF]; type of cage or housing; bedding material; number of cage companions; tank shape and material etc. for fish).<br>b. Husbandry conditions (e.g. breeding programme, light/dark cycle, temperature, quality of water etc for fish, type of food, access to food and water, environmental enrichment).<br>c. Welfare-related assessments and interventions that were carried out prior to, during, or after the experiment.	a. Conventional type, Single mouse cage with sterile bedding tip.<br>b. Giving water ad libitum in bottle, 60 kcal% fat diet, and kept at 25°C - 26°C with lights on from 7 a.m. to 7 p.m..<br>c. We changed cages and washed them once in a week.
Sample size	10	a. Specify the total number of animals used in each experiment, and the number of animals in each experimental group.<br>b. Explain how the number of animals was arrived at. Provide details of any sample size calculation used.<br>c. Indicate the number of independent replications of each experiment, if relevant.	a. 6-8 mice in a group, Total 24-32 mice.<br>b. Mice were trans ferred by car from animal laboratory company to our university. We need this number For statistical analysis.<br>c. only this time or one more time dependent on experiment.
Allocating animals to experimental groups	11	a. Give full details of how animals were allocated to experimental groups, including randomisation or matching if done.<br>b. Describe the order in which the animals in the different experimental groups were treated and assessed.	a. Active mice in a big cage were selected and allocated in order into 4 separated small cages in order individually.<br>b. Person who did not allocate to group decided the order of treatment.
Experimental outcomes	12	Clearly define the primary and secondary experimental outcomes assessed (e.g. cell death, molecular markers, behavioural changes).	Decrease body weight, Decrease adipose tissue weight, Serum parameters, Tissue parameters.
Statistical methods	13	a. Provide details of the statistical methods used for each analysis.<br>b. Specify the unit of analysis for each dataset (e.g. single animal, group of animals, single neuron).<br>c. Describe any methods used to assess whether the data met the assumptions of the statistical approach.	a. The multiple range test of Tukey in Mac Toukeikai seki Ver.2.0, Esumi, Tokyo. In some experiments, differences between 2 group data were analyzed by Student's t-test.<br>b. Group of animals.<br>c. $P < 0.05$ is significantly different.
**RESULTS**			
Baseline data	14	For each experimental group, report relevant characteristics and health status of animals (e.g. weight, microbiological status, and drug or test naïve) prior to treatment or testing. (This information can often be tabulated).	We checked health status of mice by housing before starting for one week. Abnormal mice were excluded from experiment.
Numbers analysed	15	a. Report the number of animals in each group included in each analysis. Report absolute numbers (e.g. 10/20, not 50%[2]).<br>b. If any animals or data were not included in the analysis, explain why.	a. We described number of animals used in Figure. However, we did not report absolute numbers because of confusing of drug action.<br>b. We excluded data of some mice, which fought each other and had inflammation.
Outcomes and estimation	16	Report the results for each analysis carried out, with a measure of precision (e.g. standard error or confidence interval).	Yes we did.

Adverse events	17	a. Give details of all important adverse events in each experimental group.<br>b. Describe any modifications to the experimental protocols made to reduce adverse events.	a. We cannot find adverse effect of used doses of BTS.<br>b. Higher dose (more than 3 g/kg) of BTS sometimes induces diarrhea.
**DISCUSSION**			
Interpretation/ scientific implications	18	a. Interpret the results, taking into account the study objectives and hypotheses, current theory and other relevant studies in the literature.<br>b. Comment on the study limitations including any potential sources of bias, any limitations of the animal model, and the imprecision associated with the results[2].<br>c. Describe any implications of your experimental methods or findings for the replacement, refinement or reduction (the 3Rs) of the use of animals in research.	a. We described it in the text.<br>b. Exact dosage of BTS taken into a mouse is not clear in this experimental design.<br>c. We must consider protocol of experiment such as reduction of animal number, duration of BTS administration and method of BTS treatment.
Generalisability/ translation	19	Comment on whether, and how, the findings of this study are likely to translate to other species or systems, including any relevance to human biology.	When anti-obesity mechanism of BTS is clear in mouse model, we can suppose anti-obesity action of BTS in human.
Funding	20	List all funding sources (including grant number) and the role of the funder(s) in the study.	No funding source.

References:

1. Kilkenny C, Browne WJ, Cuthill IC, Emerson M, Altman DG (2010) Improving Bioscience Research Reporting: The ARRIVE Guidelines for Reporting Animal Research. *PLoS Biol* 8(6): e1000412. doi:10.1371/journal.pbio.1000412
2. Schulz KF, Altman DG, Moher D, the CONSORT Group (2010) CONSORT 2010 Statement: updated guidelines for reporting parallel group randomised trials. *BMJ* 340:c332.

北陸大学動物実験計画書

北陸大学学長殿

■ 新規 □ 変更・年度更新

提出年月日　平成26年4月22日　受付年月日 2014年4月23日　受付番号 14－07

研究課題	防風通聖散および構成生薬の肥満症や糖尿病態に対する治療効果とその作用機序の解明

研究目的	肥満マウスや糖尿病マウスで認められる種々の病態に対する防風通聖散構成生薬の治療効果とその作用機序を解明する			
動物実験責任者名（選択項目を■）	フリガナ　コバヤシ シンジロウ	部局名	職	動物実験の経験等
	氏名 古林 伸二郎<br>e-mail s-kobayashi@hokuriku-u.ac.jp	医療薬学講座<br>連絡先TEL：076-229-1165(289)	教授	教育訓練受講の■有□無
動物実験実施者名（括弧内にフリガナ、選択項目を■）	高橋 達雄（タカハシ タツオ）<br>t-takahashi@hokuriku-u.ac.jp	医療薬学講座<br>連絡先TEL：076-229-1165(359)	講師	教育訓練受講の■有□無
	前野 洋徳（マエノ ヒロノリ）<br>2010p090@stu.hokuriku-u.ac.jp	医療薬学講座<br>連絡先TEL：076-229-1165(359)	学生(5年生)	教育訓練受講の■有□無
	池澤 賢（イケザワ ケン）<br>2007p018@stu.hokuriku-u.ac.jp	医療薬学講座<br>連絡先TEL：076-229-1165(359)	学生(5年生)	教育訓練受講の□有■無
	嶋尾 真理子（シマオ マリコ）<br>2009p061@stu.hokuriku-u.ac.jp	医療薬学講座<br>連絡先TEL：076-229-1165(359)	学生(5年生)	教育訓練受講の□有■無
	（　）<br>@	連絡先TEL：		教育訓練受講の□有□無

実験実施期間	承認後 ～ 20（ 15 ）年 3 月			中止・終了等	20（　）年　月　日	
飼養保管施設及び実験室	飼養保管施設	普通動物舎		実験室	第3飼育室	
使用動物	動物種	系統	性別	匹数	微生物学的品質	入手先（導入機関名）	備考
	マウス	ICR	雄	150	SPF	三協ラボサービス	
	マウス	ddY	雄	100	SPF	三協ラボサービス	

研究計画と方法

研究概要（研究計画と方法について、その概要を記入する。）
高脂肪食を与えて作製した肥満マウスや肥満型糖尿病態マウスに防風通聖散や構成生薬エキスを経口投与し、抗肥満作用や抗糖尿病作用とその作用機序を内臓脂肪重量や生化学的検査値測定することによって解明する。

実験方法（動物に加える処置、使用動物数の根拠を具体的に記入し、「想定される苦痛のカテゴリー」や「動物の苦痛軽減・排除方法」等と整合性をもたせる。）

マウスに高脂肪食を 5 週間自由摂取させ、肥満マウスを作製する。実験によってはマウスにエーテル麻酔下でストレプトゾトシン溶液を尾静脈内に投与した後、高脂肪食を自由摂取させ、肥満型糖尿病態マウスを作製する（想定苦痛カテゴリーB）。その後、高脂肪食自由摂取の下、防風通聖散および構成生薬エキスを 4 週間経口投与する（想定苦痛カテゴリーB）。エーテル麻酔下で頸静脈から採血を行い（想定苦痛カテゴリーB）、頸椎脱臼法により安楽死させる。体重の変化および血液生化学的検査値の測定等を行い、病勢および治療効果を判定する。

比較対照群、防風通聖散投与群、構成生薬エキス群（3つの投与量を設定）の計5群を設定し、各群につき 10 匹のマウスを用いる（5群×10 匹/群＝50 匹）。同様な実験を5回繰り返すので、動物総数が 250 匹となる。

特殊実験区分<br>(該当項目をすべて■)	□	1. 感染実験　安全度分類: □ BSL1 □ BSL2 □ BSL3
	□	2. 遺伝子組換え動物使用実験　区分: □ P1A □ P2A □ P3A
	□	3. 放射性同位元素・放射線使用実験
	□	4. 化学発癌・重金属実験

動物実験の種類<br>(選択項目を■)	■	1. 試験・研究	動物実験を<br>必要とする理由<br>(選択項目を■)	■	1. 検討したが、動物実験に替わる手段がなかった。
	□	2. 教育・訓練		□	2. 検討した代替手段の精度が不十分だった。
	□	3. その他		□	3. その他

想定される<br>苦痛のカテゴリー<br>(選択項目を■)	■	B. 脊椎動物を用い、動物に対してほとんど あるいはまったく不快感を与えないと思われる実験。
	□	C. 脊椎動物を用い、動物に対して軽度のストレスまたは痛み(短時間持続するもの)を伴うと思われる実験。
	□	D. 脊椎動物を用い、回避できない重度のストレスまたは痛み(長時間持続するもの)を伴うと思われる実験。
	□	E. 無麻酔下の脊椎動物に、耐えうる限界に近い またはそれ以上の痛みを与えると思われる実験。
動物の苦痛軽減、<br>排除の方法<br>(該当項目をすべて■)	■	1. 短時間の保定・拘束および注射など、軽微な苦痛の範囲であり、特に処置を講ずる必要はない。
	□	2. 科学上の目的を損なわない苦痛軽減方法は存在せず、処置できない。
	■	3. 麻酔薬・鎮痛薬等を使用する。<br>(具体的薬剤名及びその投与量・経路を記入:　　エーテル吸入麻酔　　)
	□	4. 動物が耐えがたい痛みを伴う場合、適切な時期に安楽死措置をとるなどの人道的エンドポイントを考慮する。
	□	5. その他 (具体的に記入:　　)
安楽死の方法<br>(該当項目をすべて■)	□	1. 麻酔薬等の使用 (具体的薬剤名及びその投与量・経路を記入:　　)
	□	2. 炭酸ガス
	■	3. 中枢破壊 (具体的に記入:　頸椎脱臼　　法)
	□	4. 安楽死させない (その理由を記入:　　)
動物死体の処理方法<br>(選択項目を■)	□	1. 大学内で焼却
	■	2. 外部業者に依託
	□	3. その他 (具体的に記入:　　)
その他必要または<br>参考事項		(過去の動物実験計画書承認実績、学内の関連委員会への申請状況、飼養保管施設・実験室の承認状況などを記入する。)

委員会記入欄	審査終了: 20(14)年 4 月 25 日
	修正意見等<br><br>古閑 健二郎 (印)
	審査結果 ☑ 本実験計画は、北陸大学における動物実験指針等に適合する。<br>(条件等 □ 遺伝子組換え実験安全委員会の承認後、実験を開始すること。)<br>□ 本実験計画は、北陸大学における動物実験指針等に適合しない。

学長承認欄	承認: 20(14)年 4 月 25 日
	本実験計画を承認します。<br><br>承認番号: 第　14-07　号<br><br>北陸大学長<br>小倉[illegible] (印)
