# Supplementary material for: Mechanisms for the anti-obesity actions of bofutsushosan in high-fat diet-fed obese mice
Source: Chin Med. 2017 Mar 27;12:8. doi: 10.1186/s13020-017-0129-x (PMC5369197; doi:10.1186/s13020-017-0129-x)
Supplement: Supplementary file 3 — Additional file 3. Summary of all datasets in Figs. 1, 2, 3, 4, 5, 6 and 7, and Table 2. [file 13020_2017_129_MOESM3_ESM.pdf]

Data of Fig. 1

6 Day	6dayコントロール	1g/kg	1.5 g/kg	2 g/kg
	6.25	−4.87	−8.68	−9.32
	−0.27	−1.62	−7.36	−8.14
	1.13	−0.29	−4.89	−6.9
	1.73	−4.06	−4.95	−5.17
	2.51	−3.54	−4.08	−10.08
	3.43	−3.61	−8.5	−10.87
		−0.81		−8.25
		−4.04		
average	2.46	−2.86	−6.41	−8.39
SD	2.24	1.70	2.01	1.94

◎ 母平均：テューキー(全群比較)

■基本統計量

カテゴリー名	n	合計	平均	分散(n−1)
6コントロール	6	14.78	2.463333333	5.012426667
1g/kg	8	−22.84	−2.855	2.891742857
1.5 g/kg	6	−38.46	−6.41	4.05888
2 g/kg	7	−58.73	−8.39	3.757

■等分散性の検定（バートレット）

分布	カイ二乗分布
自由度	3
統計量	0.428542195
1%点	11.34486673
5%点	7.814727903
p値	0.934285238
判定	[ ]

■分散分析表

要因	自由度	偏差平方和	不偏分散	分散比	p値	判定
全体	26	518.7502074				
A	3	430.6094741	143.5364914	37.4553192	5.09515E−09	[**]
誤差	23	88.14073333	3.832205797			

■母平均の差の検定と推定

カテゴリー名1	カテゴリー名2	平均1	平均2	差	統計量	5%点	1%点	判定	信頼水準95% 下限値	上限値	信頼水準99% 下限値	上限値
6コントロール	1g/kg	2.463333333	−2.855	5.318333333	7.114145459	3.9136	4.9302	[**]	2.392636993	8.244029673	1.632655672	9.004010995
6コントロール	1.5 g/kg	2.463333333	−6.41	8.873333333	11.10294043	3.9136	4.9302	[**]	5.745632384	12.00103428	4.933178177	12.81348849
6コントロール	2 g/kg	2.463333333	−8.39	10.85333333	14.09310529	3.9136	4.9302	[**]	7.839405285	13.86726138	7.056504821	14.65016185
1g/kg	1.5 g/kg	−2.855	−6.41	3.555	4.755397137	3.9136	4.9302	[*]	0.62930366	6.48069634	−0.130677662	7.240677662
1g/kg	2 g/kg	−2.855	−8.39	5.535	7.726033649	3.9136	4.9302	[**]	2.731261758	8.338738242	2.002960374	9.067039626
1.5 g/kg	2 g/kg	−6.41	−8.39	1.98	2.571039479	3.9136	4.9302	[ ]	−1.033928049	4.993928049	−1.816828512	5.776828512

12 Day	12dayコントロー	1g・kg	1.5g/kg	2g/kg
	11.01	−0.45	−6.81	−7.66
	2.49	−1.41	−7.72	−8.53
	3.67	0.94	−4.27	−5.76
	0.87	−4.71	−2.55	−2.97
	5.34	0.37	−1.42	−12.89
	5.69	−0.58	−7.06	−11.62
		4.88		−10.45
		−0.05		
average	4.85	−0.13	−4.97	−8.55
SD	3.51	2.66	2.62	3.45

◎ 母平均：テューキー(全群比較)

■基本統計量

カテゴリー名	n	合計	平均	分散(n−1)
12コントロール	6	29.07	4.845	12.33871
1g・kg	8	−1.01	−0.12625	7.060141071
1.5g/kg	6	−29.83	−4.971666667	6.853016667
2g/kg	7	−59.88	−8.554285714	11.9305619

■等分散性の検定（バートレット）

分布	カイ二乗分布
自由度	3
統計量	0.814076249
1%点	11.34486673
5%点	7.814727903
p値	0.846097303
判定	[ ]

■分散分析表

要因	自由度	偏差平方和	不偏分散	分散比	p値	判定
全体	26	877.7026				
A	3	660.7396077	220.2465359	23.34808473	3.63455E−07	[**]
誤差	23	216.9629923	9.433173577			

■母平均の差の検定と推定

カテゴリー名1	カテゴリー名2	平均1	平均2	差	統計量	5%点	1%点	判定	信頼水準95% 下限値	上限値	信頼水準99% 下限値	上限値
12コントロール	1g・kg	4.845	−0.12625	4.97125	4.23846196	3.9136	4.9302	[*]	0.381027372	9.561472628	−0.811332687	10.75383269
12コントロール	1.5g/kg	4.845	−4.971666667	9.816666667	7.829080466	3.9136	4.9302	[**]	4.909512265	14.72382107	3.634825745	15.99850759
12コントロール	2g/kg	4.845	−8.554285714	13.39928571	11.08972308	3.9136	4.9302	[**]	8.670633413	18.12793802	7.442314748	19.35625668
1g・kg	1.5g/kg	−0.12625	−4.971666667	4.845416667	4.131177113	3.9136	4.9302	[*]	0.255194038	9.435639295	−0.93716602	10.62799935
1g・kg	2g/kg	−0.12625	−8.554285714	8.428035714	7.498265884	3.9136	4.9302	[**]	4.029157216	12.82691421	2.886500869	13.96957056
1.5g/kg	2g/kg	−4.971666667	−8.554285714	3.582619048	2.965102319	3.9136	4.9302	[ ]	−1.146033253	8.311271348	−2.374351919	9.539590014

18 Day	18dayコントロー	1g/kg	1.5g/kg	2g/kg
	14.07	4.3	−3.48	−6.72
	9.47	0.84	−2.32	−7.5
	6.68	4.05	−0.73	−3.95
	0.45	−1.5	−1.67	−1.14
	4.07	3.63	0.7	−10.73
	7.32	0.01	−3.44	−8.53
		8.47		−7.43
		6.3		
average	7.01	3.26	−1.82	−6.57
SD	4.64	3.32	1.62	3.14

◎ 母平均：テューキー(全群比較)

■基本統計量

カテゴリー名	n	合計	平均	分散(n−1)
18コントロール	6	42.06	7.01	21.55548
1g/kg	8	26.1	3.2625	11.04353571
1.5g/kg	6	−10.94	−1.823333333	2.638186667
2g/kg	7	−46	−6.571428571	9.853914286

■等分散性の検定（バートレット）

分布	カイ二乗分布
自由度	3
統計量	4.473077091
1%点	11.34486673
5%点	7.814727903
p値	0.214704358
判定	[ ]

■分散分析表

要因	自由度	偏差平方和	不偏分散	分散比	p値	判定
全体	26	954.9588667				
A	3	697.5622976	232.5207659	20.77719076	9.69964E−07	[**]
誤差	23	257.396569	11.19115518			

■母平均の差の検定と推定

カテゴリー名1	カテゴリー名2	平均1	平均2	差	統計量	5%点	1%点	判定	信頼水準95% 下限値	上限値	信頼水準99% 下限値	上限値
18コントロール	1g/kg	7.01	3.2625	3.7475	2.933430352	3.9136	4.9302	[ ]	−1.252181001	8.747181001	−2.550902307	10.04590231
18コントロール	1.5g/kg	7.01	−1.823333333	8.833333333	6.467892388	3.9136	4.9302	[**]	3.48844952	14.17821715	2.100058043	15.56660862
18コントロール	2g/kg	7.01	−6.571428571	13.58142857	10.31991124	3.9136	4.9302	[**]	8.430969655	18.73188749	7.093082151	20.06977499
1g/kg	1.5g/kg	3.2625	−1.823333333	5.085833333	3.981037456	3.9136	4.9302	[*]	0.086152333	10.08551433	−1.212568974	11.38423564
1g/kg	2g/kg	3.2625	−6.571428571	9.833928571	8.032541397	3.9136	4.9302	[**]	5.042660036	14.62519711	3.798076125	15.86978102
1.5g/kg	2g/kg	−1.823333333	−6.571428571	4.748095238	3.6078621	3.9136	4.9302	[ ]	−0.402363679	9.898554155	−1.740251182	11.23644166

25 Day	25dayコントロー	1g/kg	1.5g/kg	2g/kg
	19.13	16.19	7.39	1.45
	19.02	4.95	9.57	−1.01
	8.8	11.97	4.21	2.71
	5.28	5.73	5.48	5.08
	7.2	9.59	6.98	−0.8
	12.15	4.34	5.98	3.81
		13.11		3.54
		14.45		
average	11.93	10.04	6.60	2.11
SD	5.98	4.59	1.84	2.34

◎ 母平均：テューキー(全群比較)

■基本統計量

カテゴリー名	n	合計	平均	分散(n−1)
25コントロール	6	71.58	11.93	35.70976
1g/kg	8	80.33	10.04125	21.08529821
1.5g/kg	6	39.61	6.601666667	3.388056667
2g/kg	7	14.78	2.111428571	5.458980952

■等分散性の検定（バートレット）

分布	カイ二乗分布
自由度	3
統計量	8.14930028
1%点	11.34486673
5%点	7.814727903
p値	0.043024818
判定	[*]

■分散分析表

要因	自由度	偏差平方和	不偏分散	分散比	p値	判定
全体	26	752.8171852				
A	3	376.9771286	125.6590429	7.689861514	0.000984939	[**]
誤差	23	375.8400565	16.34087202			

■母平均の差の検定と推定

カテゴリー名1	カテゴリー名2	平均1	平均2	差	統計量	5%点	1%点	判定	信頼水準95% 下限値	上限値	信頼水準99% 下限値	上限値
25コントロール	1g/kg	11.93	10.04125	1.88875	1.223512644	3.9136	4.9302	[ ]	−4.152717604	7.930217604	−5.722054268	9.499554268
25コントロール	1.5g/kg	11.93	6.601666667	5.328333333	3.228712661	3.9136	4.9302	[ ]	−1.130267206	11.78693387	−2.807958669	13.46462534
25コントロール	2g/kg	11.93	2.111428571	9.818571429	6.174171041	3.9136	4.9302	[**]	3.594908221	16.04223464	1.978244275	17.65889858
1g/kg	1.5g/kg	10.04125	6.601666667	3.439583333	2.228126378	3.9136	4.9302	[ ]	−2.601884271	9.481050938	−4.171220935	11.0503876
1g/kg	2g/kg	10.04125	2.111428571	7.929821429	5.360300968	3.9136	4.9302	[**]	2.140193323	13.71944953	0.636274697	15.22336816
1.5g/kg	2g/kg	6.601666667	2.111428571	4.490238095	2.823577566	3.9136	4.9302	[ ]	−1.733425112	10.7139013	−3.350089058	12.33056525

Body Weight

25 Day	25dayコントロー	1g/kg	1.5g/kg	2g/kg
	65.22	55.92	50.22	43.9
	72.43	49.36	46.03	44.71
	56.54	64.83	43.95	39.63
	49.7	58.39	49.4	50.92
	47.62	47.18	55.43	46.58
	62.17	44.24	42.95	45.62
		68.36		49.85
		59.05		
average	58.95	55.92	48.00	45.89
SD	9.50	8.50	4.64	3.79

◎ 母平均：テューキー(全群比較)

■基本統計量

カテゴリー名	n	合計	平均	分散(n−1)
25dayコントロー	6	353.68	58.94666667	90.22622667
1g/kg	8	447.33	55.91625	72.26899821
1.5g/kg	6	287.98	47.99666667	21.57582667
2g/kg	7	321.21	45.88714286	14.34525714

■等分散性の検定（バートレット）

分布	カイ二乗分布
自由度	3
統計量	5.757017127
1%点	11.34486673
5%点	7.814727903
p値	0.124049236
判定	[ ]

■分散分析表

要因	自由度	偏差平方和	不偏分散	分散比	p値	判定
全体	26	1919.503496				
A	3	768.5386993	256.1795664	5.119296475	0.007369245	[**]
誤差	23	1150.964797	50.0419477			

■母平均の差の検定と推定

カテゴリー名1	カテゴリー名2	平均1	平均2	差	統計量	5%点	1%点	判定	信頼水準95% 下限値	上限値	信頼水準99% 下限値	上限値
25dayコントロー	1g/kg	58.94666667	55.91625	3.030416667	1.121777803	3.9136	4.9302	[ ]	−7.541943238	13.60277657	−10.28822826	16.3490616
25dayコントロー	1.5g/kg	58.94666667	47.99666667	10.95	3.791601113	3.9136	4.9302	[ ]	−0.352328153	22.25232815	−3.28823034	25.18823034
25dayコントロー	2g/kg	58.94666667	45.88714286	13.05952381	4.692758511	3.9136	4.9302	[*]	2.168327884	23.95071974	−0.660778253	26.77982587
1g/kg	1.5g/kg	55.91625	47.99666667	7.919583333	2.931614286	3.9136	4.9302	[ ]	−2.652776572	18.49194324	−5.399061598	21.23822826
1g/kg	2g/kg	55.91625	45.88714286	10.02910714	3.873990492	3.9136	4.9302	[ ]	−0.102542327	20.16075661	−2.734348044	22.79256233
1.5g/kg	2g/kg	47.99666667	45.88714286	2.10952381	0.758028084	3.9136	4.9302	[ ]	−8.781672116	13.00071974	−11.61077825	15.82982587

Data of Fig. 2

Retroperitoneal				
	高脂肪食	高脂肪食	高脂肪食	高脂肪食
	コントロール	1,000mg/kg	1,500mg/kg	2000mg/kg
	2.5805	2.4525	1.2109	0.7687
	2.617	1.2901	1.9385	1.2825
	3.5383	3.4211	0.5472	0.6825
	2.9315	1.8462	1.4245	1.7183
	3.0805	0.7109	2.5675	1.7681
	2.8747	0.8682	0.5407	1.2548
		4.1607		0.9318
Average	2.937	2.107	1.372	1.201
SD	0.351	1.309	0.794	0.433
	0.124	0.463	0.300	0.164
	t-test	0.178658615	0.001296694	7.90641E-06

Epididymal				
	高脂肪食	高脂肪食	高脂肪食	高脂肪食
	コントロール	1,000mg/kg	1,500mg/kg	2000mg/kg
	5.882	4.7448	1.752	1.8756
	2.6098	1.5024	2.0087	1.6801
	3.535	5.2725	1.3615	0.86
	4.1219	1.4687	2.7516	2.3967
	3.2372	1.9438	1.029	2.2731
	3.4804	3.1446		
Average	3.81105	3.0128	1.78056	1.8171
SD	1.126348084	1.66923655	0.658831529	0.608708432
	0.398224184	0.590164242	0.268966845	0.230070162
	t-test	0.364172127	0.010449446	0.010518622

Mesenteric				
	高脂肪食	高脂肪食	高脂肪食	高脂肪食
	コントロール	1,000mg/kg	1,500mg/kg	2000mg/kg
	1.56	1.8404	0.5159	0.4782
	0.8377	0.4427	0.6114	0.5996
	1.0798	1.0555	0.2501	0.2126
	0.9953	0.8726	0.524	0.6011
	0.7102	0.317	0.9287	0.4957
	1.2284	0.3133	0.3478	0.5065
		1.5137		0.557
Average	1.068566667	0.907885714	0.52965	0.492957143
SD	0.301421198	0.602592282	0.235526039	0.133027502
	0.106568487	0.213048544	0.096153103	0.05027967
	t-test	0.373614821	0.006216611	0.000785681

Periretinal				
	高脂肪食	高脂肪食	高脂肪食	高脂肪食
	コントロール	1,000mg/kg	1,500mg/kg	2000mg/kg
	1.5074	1.3282	0.4125	0.247
	0.852	0.3741	0.729	0.1112
	1.2771	1.2243	0.4713	0.2501
	0.7117	0.8752	0.6287	0.7516
	0.961	0.5945	0.7919	0.4356
	1.1354	0.571	0.2585	0.6093
				0.5963
Average	1.0741	0.827883333	0.54865	0.428728571
SD	0.291948406	0.383686838	0.203377263	0.234782607
	0.103219349	0.135653783	0.08302842	0.088739484
	t-test	0.183694714	0.004709919	0.001024781

Total WAT				
	高脂肪食	高脂肪食	高脂肪食	高脂肪食
	コントロール	1,000mg/kg	1,500mg/kg	2000mg/kg
	11.53	10.3659	3.8913	3.3695
	6.9165	3.6093	5.2876	3.6734
	9.4302	10.9734	2.6301	2.0052
	8.7604	4.2264	5.3171	5.4677
	7.9889	3.5662	1.147	3.33047567
	8.7189	4.8971		
Average	8.890816667	6.27305	3.65462	3.569255134
SD	1.550107536	3.445267097	1.790550205	1.240778513
	0.548045775	1.218085864	0.73098906	0.468970197
	t-test	0.154179469	0.001477519	0.000495994

◎ 母平均:ｼｪｯﾌｪ(全群比較)

◎ バートレットの検定

項目	件数	分散(n-1)
コントロール	6	0.123111538
1,000mg/kg	7	1.713829263
1,500mg/kg	6	0.630041095
2000mg/kg	7	0.187822093

分布	χ2分布
統計量	10.60418914
自由度	3
5%点	7.814727903
1%点	11.34486673
確率	0.014070525
検定マーク	[*]

◎ 平均値表

項目名	コントロール	1,000mg/kg	1,500mg/kg	2000mg/kg	全体
件数	6	7	6	7	26
データ計	17.6225	14.7497	8.2293	8.4067	49.0082
平均値	2.937083333	2.1071	1.37155	1.200957143	1.884930769

◎ 分散分析表

変動因子	自由度	偏差平方和	不偏分散	分散比	p値	判定
全体(T)	25	27.01943392				
因子(A)	3	11.84376261	3.947920872	5.723256484	0.004723291	[**]
誤差(E)	22	15.1756713	0.689803241			

◎ 同時信頼域表

同時信頼域表			0.05			0.01			
項目名	項目名	平均値差	精度	下限値	上限値	精度	下限値	上限値	判定
コントロール	1,000mg/kg	0.829983333	1.397519534	-0.567536201	2.227502868	1.756470357	-0.926487023	2.58645369	[ ]
コントロール	1,500mg/kg	1.565533333	1.45027456	0.115258773	3.015807893	1.822775432	-0.257242099	3.388308765	[*]
コントロール	2000mg/kg	1.73612619	1.397519534	0.338606656	3.133645725	1.756470357	-0.020344166	3.492596547	[*]
1,000mg/kg	1,500mg/kg	0.73555	1.397519534	-0.661969534	2.133069534	1.756470357	-1.020920357	2.492020357	[ ]
1,000mg/kg	2000mg/kg	0.906142857	1.342693338	-0.436550481	2.248836195	1.687562132	-0.781419275	2.59370499	[ ]
1,500mg/kg	2000mg/kg	0.170592857	1.397519534	-1.226926677	1.568112392	1.756470357	-1.585877499	1.927063214	[ ]

◎ 母平均:ﾃｭｰｷｰ(全群比較)

■基本統計量

カテゴリー名	n	合計	平均	分散(n-1)
コントロール	6	6.4446	1.0741	0.085233872
1,000mg/kg	6	4.9673	0.827883333	0.14721559
1,500mg/kg	6	3.2919	0.54865	0.041362311
2000mg/kg	7	3.0011	0.428728571	0.055122872

■等分散性の検定(バートレット)

分布	カイ二乗分布
自由度	3
統計量	2.234329981
1%点	11.34486673
5%点	7.814727903
p値	0.525218127
判定	[ ]

■分散分析表

要因	自由度	偏差平方和	不偏分散	分散比	p値	判定
全体	24	3.28850473				
A	3	1.588708632	0.529569544	6.542526153	0.002690259	[**]
誤差	21	1.699796098	0.080942671			

■母平均の差の検定と推定

									信頼水準95%		信頼水準99%	
カテゴリー名1	カテゴリー名2	平均1	平均2	差	統計量	5%点	1%点	判定	下限値	上限値	下限値	上限値
コントロール	1,000mg/kg	1.0741	0.827883333	0.246216667	2.119845971	3.9419	4.9856	[ ]	-0.211628616	0.70406195	-0.332852676	0.825286009
コントロール	1,500mg/kg	1.0741	0.54865	0.52545	4.523954778	3.9419	4.9856	[*]	0.067604717	0.983295283	-0.053619343	1.104519343
コントロール	2000mg/kg	1.0741	0.428728571	0.645371429	5.76618982	3.9419	4.9856	[**]	0.204180675	1.086562183	0.087366248	1.20337661
1,000mg/kg	1,500mg/kg	0.827883333	0.54865	0.279233333	2.404108807	3.9419	4.9856	[ ]	-0.17861195	0.737078616	-0.299836009	0.858302676
1,000mg/kg	2000mg/kg	0.827883333	0.428728571	0.399154762	3.566321691	3.9419	4.9856	[ ]	-0.042035992	0.840345516	-0.158850419	0.957159943
1,500mg/kg	2000mg/kg	0.54865	0.428728571	0.119921429	1.071460077	3.9419	4.9856	[ ]	-0.321269325	0.561112183	-0.438083752	0.67792661

◎ 母平均:ﾃｭｰｷｰ(全群比較)

■基本統計量

カテゴリー名	n	合計	平均	分散(n-1)
11.53	5	41.8149	8.36298	0.913955117
10.3659	5	27.2724	5.45448	9.811905377
3.8913	4	14.3818	3.59545	4.251419457
3.3695	4	14.47677567	3.619193918	2.036082543

■等分散性の検定(バートレット)

分布	カイ二乗分布
自由度	3
統計量	4.930756784
1%点	11.34486673
5%点	7.814727903
p値	0.176938259
判定	[ ]

■分散分析表

要因	自由度	偏差平方和	不偏分散	分散比	p値	判定
全体	17	131.3568175				
A	3	69.59086953	23.19695651	5.257871073	0.012240235	[*]
誤差	14	61.76594797	4.411853427			

■母平均の差の検定と推定

									信頼水準95%		信頼水準99%	
カテゴリー名1	カテゴリー名2	平均1	平均2	差	統計量	5%点	1%点	判定	下限値	上限値	下限値	上限値
11.53	10.3659	8.36298	5.45448	2.9085	3.096303563	4.1105	5.3215	[ ]	-0.952681246	6.769681246	-2.090229109	7.907229109
11.53	3.8913	8.36298	3.59545	4.76753	4.785106826	4.1105	5.3215	[*]	0.672128836	8.862931164	-0.534422876	10.06948288
11.53	3.3695	8.36298	3.619193918	4.743786083	4.76127537	4.1105	5.3215	[*]	0.648384918	8.839187247	-0.558166793	10.04573896
10.3659	3.8913	5.45448	3.59545	1.85903	1.865883831	4.1105	5.3215	[ ]	-2.236371164	5.954431164	-3.442922876	7.160982876
10.3659	3.3695	5.45448	3.619193918	1.835286083	1.842052375	4.1105	5.3215	[ ]	-2.260115082	5.930687247	-3.466666793	7.137238958
3.8913	3.3695	3.59545	3.619193918	-0.023743918	0.022608504	4.1105	5.3215	[ ]	-4.340675788	4.293187953	-5.612492962	5.565005127

◎ 母平均:ﾃｭｰｷｰ(全群比較)

■基本統計量

カテゴリー名	n	合計	平均	分散(n-1)
コントロール	6	22.8663	3.81105	1.268660007
1,000mg/kg	6	18.0768	3.0128	2.78635066
1,500mg/kg	5	8.9028	1.78056	0.434058983
2000mg/kg	5	9.0855	1.8171	0.370525955

■等分散性の検定(バートレット)

分布	カイ二乗分布
自由度	3
統計量	5.293716897
1%点	11.34486673
5%点	7.814727903
p値	0.151510612
判定	[ ]

■分散分析表

要因	自由度	偏差平方和	不偏分散	分散比	p値	判定
全体	21	39.60147918				
A	3	16.10808609	5.369362031	4.113859424	0.021862271	[*]
誤差	18	23.49339309	1.305188505			

■母平均の差の検定と推定

									信頼水準95%		信頼水準99%	
カテゴリー名1	カテゴリー名2	平均1	平均2	差	統計量	5%点	1%点	判定	下限値	上限値	下限値	上限値
コントロール	1,000mg/kg	3.81105	3.0128	0.79825	1.711504055	3.997	5.0941	[ ]	-1.06596133	2.66246133	-1.57765166	3.17415166
コントロール	1,500mg/kg	3.81105	1.78056	2.03049	4.150911915	3.997	5.0941	[*]	0.075288662	3.985691338	-0.461376684	4.522356684
コントロール	2000mg/kg	3.81105	1.8171	1.99395	4.076213531	3.997	5.0941	[*]	0.038748662	3.949151338	-0.497916684	4.485816684
1,000mg/kg	1,500mg/kg	3.0128	1.78056	1.23224	2.519056828	3.997	5.0941	[ ]	-0.722961338	3.187441338	-1.259626684	3.724106684
1,000mg/kg	2000mg/kg	3.0128	1.8171	1.1957	2.444358444	3.997	5.0941	[ ]	-0.759501338	3.150901338	-1.296166684	3.687566684
1,500mg/kg	2000mg/kg	1.78056	1.8171	-0.03654	0.071518258	3.997	5.0941	[ ]	-2.078681195	2.005601195	-2.639209868	2.566129868

◎ 母平均:ｼｪｯﾌｪ(全群比較)

◎ バートレットの検定

項目	件数	分散(n-1)
コントロール	6	0.090854739
1,000mg/kg	7	0.363117458
1,500mg/kg	6	0.055472515
2000mg/kg	7	0.017696316

分布	χ2分布
統計量	12.09897706
自由度	3
5%点	7.814727903
1%点	11.34486673
確率	0.007051684
検定マーク	[**]

◎ 平均値表

項目名	コントロール	1,000mg/kg	1,500mg/kg	2000mg/kg	全体
件数	6	7	6	7	26
データ計	6.4114	6.3552	3.1779	3.4507	19.3952
平均値	1.068566667	0.907885714	0.52965	0.492957143	0.745969231

◎ 分散分析表

変動因子	自由度	偏差平方和	不偏分散	分散比	p値	判定
全体(T)	25	4.553322055				
因子(A)	3	1.536803141	0.512267714	3.736058027	0.02610236	[*]
誤差(E)	22	3.016518914	0.137114496			

◎ 同時信頼域表

同時信頼域表			0.05			0.01			
項目名	項目名	平均値差	精度	下限値	上限値	精度	下限値	上限値	判定
コントロール	1,000mg/kg	0.160680952	0.623070168	-0.462389215	0.78375112	0.783104817	-0.622423865	0.943785769	[ ]
コントロール	1,500mg/kg	0.538916667	0.64659047	-0.107673804	1.185507137	0.812666275	-0.273749608	1.351582941	[ ]
コントロール	2000mg/kg	0.575609524	0.623070168	-0.047460644	1.198679691	0.783104817	-0.207495293	1.358714341	[ ]
1,000mg/kg	1,500mg/kg	0.378235714	0.623070168	-0.244834453	1.001305882	0.783104817	-0.404869103	1.161340531	[ ]
1,000mg/kg	2000mg/kg	0.414928571	0.598626454	-0.183697882	1.013555025	0.752382772	-0.3374542	1.167311343	[ ]
1,500mg/kg	2000mg/kg	0.036692857	0.623070168	-0.58637731	0.659763025	0.783104817	-0.74641196	0.819797674	[ ]

Data of Fig. 3

Total Fat Mass (cm2)		Control	With BTS
		14.49	2.05
		9.87	0.90
		6.37	3.32
	Average	10.24	2.09
	SD	4.07	1.21
	t-test		#NAME?
Visceral Fat Mass (cm2)		Control	With BTS
		9.89	1.22
		5.66	0.46
		4.90	2.48
	Average	6.82	1.38
	SD	2.69	1.02
	t-test		#NAME?
Body Fat %		Control	With BTS
		47.15	11.863
		34.289	7.325
		27.822	16.473
	Average	36.42	11.89
	SD	9.84	4.57
	t-test		#NAME?

Data of Fig. 4

Size of Adipocyte (micro m2)			
		Control	plus BTS
14 days		6655	2468
25 days		7104	1509
		13107	3928
		10555	2210
	Average	10255	2549
	SD	3013	1245
	t-test		0.01491487

Data of Fig. 5

	Glucose			
	コントロール	1	1.5	2
	126	182	155	89
	172	135	164	95
	140	197	143	119
	213	190	99	116
	146	160	137	105
	158	95	100	111
Average	159.2	159.8	133.0	105.8
SD	30.7	39.0	27.6	11.9
	Insulin			
	コントロール	1	1.5	2
	16.93	16.73	4.26	1.05
	13.60	4.28	5.67	3.27
	8.54	7.14	4.49	2.23
	10.04	6.84	2.34	3.53
	12.30	19.76	6.38	2.23
	16.82	12.34	1.02	3.12
Average	13.04	11.18	4.03	2.57
SD	3.45	6.14	2.02	0.92
	HOMA-IR			
	コントロール	1	1.5	2
	136.9	195.4	42.4	6.0
	150.2	37.1	59.7	19.9
	76.8	90.3	41.3	17.0
	137.3	83.5	14.9	26.3
	115.3	203.0	56.1	15.0
	170.6	75.3	6.6	22.3
Average	131.18	114.08	36.82	17.76
SD	32.23	68.49	21.66	6.98

◎ 母平均：ﾃｭｰｷｰ(全群比較)

■基本統計量

カテゴリー名	n	合計	平均	分散(n-1)
コントロール	6	955	159.1666667	940.9666667
1	6	959	159.8333333	1520.566667
1.5	6	798	133	761.2
2	6	635	105.8333333	140.9666667

■等分散性の検定（バートレット）

分布	カイ二乗分布
自由度	3
統計量	5.450050373
1%点	11.34486673
5%点	7.814727903
p値	0.14165687
判定	[ ]

■分散分析表

要因	自由度	偏差平方和	不偏分散	分散比	p値	判定
全体	23	28673.95833				
A	3	11855.45833	3951.819444	4.699372054	0.012167924	[*]
誤差	20	16818.5	840.925			

■母平均の差の検定と推定

									信頼水準95%		信頼水準99%	
カテゴリー名1	カテゴリー名2	平均1	平均2	差	統計量	5%点	1%点	判定	下限値	上限値	下限値	上限値
コントロール	1	159.1666667	159.8333333	-0.666666667	0.05631262	3.9583	5.0178	[ ]	-47.52768404	46.19435071	-60.07075756	58.73742423
コントロール	1.5	159.1666667	133	26.16666667	2.210270337	3.9583	5.0178	[ ]	-20.69435071	73.02768404	-33.23742423	85.57075756
コントロール	2	159.1666667	105.8333333	53.33333333	4.505009604	3.9583	5.0178	[*]	6.472315959	100.1943507	-6.070757559	112.7374242
1	1.5	159.8333333	133	26.83333333	2.266582957	3.9583	5.0178	[ ]	-20.02768404	73.69435071	-32.57075756	86.23742423
1	2	159.8333333	105.8333333	54	4.561322224	3.9583	5.0178	[*]	7.138982626	100.8610174	-5.404090893	113.4040909
1.5	2	133	105.8333333	27.16666667	2.294739267	3.9583	5.0178	[ ]	-19.69435071	74.02768404	-32.23742423	86.57075756

◎ 母平均：ｼｪｯﾌｪ(全群比較)

◎ バートレットの検定

項目	件数	分散(n-1)
コントロール	6	11.88946789
1	6	37.69618021
1.5	6	4.082746769
2	6	0.850606803

分布	χ2分布
統計量	14.30235379
自由度	3
5%点	7.814727903
1%点	11.34486673
確率	0.002521193
検定マーク	[**]

◎ 平均値表

項目名	コントロール	1	1.5	2	全体
件数	6	6	6	6	24
データ計	78.23643076	67.08667223	24.16785903	15.43257753	184.9235395
平均値	13.03940513	11.18111204	4.027976505	2.572096255	7.705147481

◎ 分散分析表

変動因子	自由度	偏差平方和	不偏分散	分散比	p値	判定
全体(T)	23	755.0336214				
因子(A)	3	482.4386131	160.812871	11.79866587	0.000114089	[**]
誤差(E)	20	272.5950084	13.62975042			

◎ 同時信頼域表

同時信頼域表			0.05			0.01			
項目名	項目名	平均値差	精度	下限値	上限値	精度	下限値	上限値	判定
コントロール	1	1.85829309	6.498484355	-4.640191266	8.356777445	8.204044327	-6.345751237	10.06233742	[ ]
コントロール	1.5	9.011428622	6.498484355	2.512944267	15.50991298	8.204044327	0.807384295	17.21547295	[**]
コントロール	2	10.46730887	6.498484355	3.968824517	16.96579323	8.204044327	2.263264546	18.6713532	[**]
1	1.5	7.153135532	6.498484355	0.654651177	13.65161989	8.204044327	-1.050908794	15.35717986	[*]
1	2	8.609015783	6.498484355	2.110531427	15.10750014	8.204044327	0.404971456	16.81306011	[**]
1.5	2	1.45588025	6.498484355	-5.042604105	7.954364606	8.204044327	-6.748164076	9.659924577	[ ]

◎ 母平均：ｼｪｯﾌｪ(全群比較)

◎ バートレットの検定

項目	件数	分散(n-1)
コントロール	6	1038.86443
1	6	4691.46544
1.5	6	468.9536567
2	6	48.74117217

分布	χ2分布
統計量	18.36227682
自由度	3
5%点	7.814727903
1%点	11.34486673
確率	0.000370299
検定マーク	[**]

◎ 平均値表

項目名	コントロール	1	1.5	2	全体
件数	6	6	6	6	24
データ計	787.0885037	684.493	220.91	106.543	1799.034504
平均値	131.1814173	114.0821667	36.81833333	17.75716667	74.95977099

◎ 分散分析表

変動因子	自由度	偏差平方和	不偏分散	分散比	p値	判定
全体(T)	23	87750.17888				
因子(A)	3	56510.05538	18836.68513	12.0592898	9.95065E-05	[**]
誤差(E)	20	31240.1235	1562.006175			

◎ 同時信頼域表

同時信頼域表			0.05			0.01			
項目名	項目名	平均値差	精度	下限値	上限値	精度	下限値	上限値	判定
コントロール	1	17.09925062	69.56799699	-52.46874637	86.66724761	87.82646842	-70.72721781	104.925719	[ ]
コントロール	1.5	94.36308395	69.56799699	24.79508696	163.9310809	87.82646842	6.536615527	182.1895524	[**]
コントロール	2	113.4242506	69.56799699	43.85625363	182.9922476	87.82646842	25.59778219	201.250719	[**]
1	1.5	77.26383333	69.56799699	7.695836341	146.8318303	87.82646842	-10.56263509	165.0903018	[*]
1	2	96.325	69.56799699	26.75700301	165.892997	87.82646842	8.498531576	184.1514684	[**]
1.5	2	19.06116667	69.56799699	-50.50683033	88.62916366	87.82646842	-68.76530176	106.8876351	[ ]

Data of Fig. 6

Leptin	コントロール	1.0g/kg	1,5g/kg	2.0 g/kg
	79.144	85.470	7.384	3.775
	82.254	63.756	10.975	6.063
	44.143	30.377	6.923	4.138
	13.346	8.864	8.253	11.979
	48.192	82.156	31.342	11.211
	71.219	44.080	1.669	11.329
				12.068
Average	56.383	52.450	11.091	8.652
SD	26.369	30.191	10.374	3.815

Adiponectin	コントロール	1.0g/kg	1,5g/kg	2.0 g/kg
	12.107	10.477	9.480	10.379
	13.503	10.100	11.998	10.477
	11.925	12.049	10.964	10.100
	11.651	7.105	8.436	12.049
	9.018	10.470	7.322	10.843
	6.609	12.114	7.322	9.699
				9.761
Average	10.802	10.386	9.254	10.473
SD	2.520	1.823	1.931	0.804

◎ 母平均：ｼｪｯﾌｪ(全群比較)

◎ バートレットの検定

項目	件数	分散(n−1)
コントロール	6	695.3238433
1.0g/kg	6	911.5236358
1,5g/kg	6	107.6141208
2.0 g/kg	7	14.55270015

分布	χ2分布
統計量	18.70840041
自由度	3
5%点	7.814727903
1%点	11.34486673
確率	0.0003141
検定マーク	[**]

◎ 平均値表

項目名	コントロール	1.0g/kg	1,5g/kg	2.0 g/kg	全体
件数	6	6	6	7	25
データ計	338.2979782	314.7029687	66.54614957	60.56326485	780.1103613
平均値	56.38299636	52.45049479	11.09102493	8.651894978	31.20441445

◎ 分散分析表

変動因子	自由度	偏差平方和	不偏分散	分散比	p値	判定
全体(T)	24	21159.36927				
因子(A)	3	12499.74507	4166.58169	10.1041585	0.000252526	[**]
誤差(E)	21	8659.6242	412.3630572			

◎ 同時信頼域表

同時信頼域表			0.05			0.01			
項目名	項目名	平均値差	精度	下限値	上限値	精度	下限値	上限値	判定
コントロール	1.0g/kg	3.932501574	35.59454845	−31.66204688	39.52705003	44.8316472	−40.89914562	48.76414877	[ ]
コントロール	1,5g/kg	45.29197143	35.59454845	9.697422982	80.88651989	44.8316472	0.460324236	90.12361863	[**]
コントロール	2.0 g/kg	47.73110138	34.29976513	13.43133625	82.03086652	43.20085621	4.530245178	90.93195759	[**]
1.0g/kg	1,5g/kg	41.35946986	35.59454845	5.764921408	76.95401831	44.8316472	−3.472177338	86.19111706	[*]
1.0g/kg	2.0 g/kg	43.79859981	34.29976513	9.498834678	78.09836494	43.20085621	0.597743605	86.99945602	[**]
1,5g/kg	2.0 g/kg	2.43912995	34.29976513	−31.86063518	36.73889508	43.20085621	−40.76172626	45.63998616	[ ]

◎ 母平均：ﾃｭｰｷｰ(全群比較)

■基本統計量

カテゴリー名	n	合計	平均	分散(n−1)
コントロール	6	64.81294884	10.80215814	6.349190095
1.0g/kg	6	62.31588384	10.38598064	3.32227489
1,5g/kg	6	55.52170225	9.253617041	3.727717966
2.0 g/kg	7	73.30881683	10.47268812	0.645655431

■等分散性の検定（バートレット）

分布	カイ二乗分布
自由度	3
統計量	5.87450577
1%点	11.34486673
5%点	7.814727903
p値	0.117878005
判定	[ ]

■分散分析表

要因	自由度	偏差平方和	不偏分散	分散比	p値	判定
全体	24	79.11048836				
A	3	8.240641018	2.746880339	0.813949646	0.500476627	[ ]
誤差	21	70.86984734	3.374754635			

■母平均の差の検定と推定

									信頼水準95%		信頼水準99%	
カテゴリー名1	カテゴリー名2	平均1	平均2	差	統計量	5%点	1%点	判定	下限値	上限値	下限値	上限値
コントロール	1.0g/kg	10.80215814	10.38598064	0.4161775	0.554923506	3.9419	4.9856	[ ]	−2.540140031	3.372495031	−3.322886576	4.155241577
コントロール	1,5g/kg	10.80215814	9.253617041	1.5485411	2.064796523	3.9419	4.9856	[ ]	−1.407776431	4.50485863	−2.190522977	5.287605176
コントロール	2.0 g/kg	10.80215814	10.47268812	0.329470022	0.455892839	3.9419	4.9856	[ ]	−2.519308833	3.178248876	−3.273582277	3.932522321
1.0g/kg	1,5g/kg	10.38598064	9.253617041	1.132363599	1.509873018	3.9419	4.9856	[ ]	−1.823953932	4.08868113	−2.606700477	4.871427675
1.0g/kg	2.0 g/kg	10.38598064	10.47268812	−0.086707479	0.119978499	3.9419	4.9856	[ ]	−2.935486333	2.762071376	−3.689759778	3.51634482
1,5g/kg	2.0 g/kg	9.253617041	10.47268812	−1.219071078	1.686847778	3.9419	4.9856	[ ]	−4.067849932	1.629707777	−4.822123377	2.383981221

Data of Fig. 7

Triglycerol	コントロール	1g/kg	1.5g/kg	2 g/kg
	3.8491	1.4859	1.6232	1.7176
	2.0532	1.9494	0.6362	0.9709
	3.6214	2.5416	1.7244	2.1038
	2.1291	1.4714	0.6877	1.7691
	2.6400	1.7691	1.4859	0.5589
	1.5889	2.5674	0.9194	1.4859
Average	2.6469	1.9641	1.1795	1.4344
SD	0.9094	0.4913	0.4883	0.5700

HDL	コントロール	1g/kg	1.5g/kg	2 g/kg
	1.912	2.088	2.388	1.695
	2.124	2.073	1.868	1.783
	1.956	1.849	2.011	2.267
	1.541	2.267	1.952	2.429
	1.703	2.022	2.355	1.805
	1.967	1.633	1.541	1.809
Average	1.8672	1.9888	2.0194	1.9650
SD	0.2093	0.2198	0.3178	0.3039

LDL	コントロール	1g/kg	1.5g/kg	2 g/kg
	0.354	0.379	0.456	0.401
	0.339	0.296	0.250	0.427
	0.394	0.447	0.317	0.419
	0.317	0.329	0.337	0.435
	0.296	0.266	0.337	0.354
	0.274	0.427	0.227	0.223
Average	0.3289	0.3573	0.3207	0.3767
SD	0.0430	0.0726	0.0806	0.0806

◎ 母平均:テューキー(全群比較)

■基本統計量

カテゴリー名	n	合計	平均	分散(n-1)
コントロール	6	15.881681	2.646946833	0.826996291
1g/kg	6	11.784793	1.964132167	0.241409096
1.5g/kg	6	7.076742	1.179457	0.238476421
2 g/kg	6	8.606291	1.434381833	0.324854236

■等分散性の検定(バートレット)

分布	カイ二乗分布
自由度	3
統計量	2.688376049
1%点	11.34486673
5%点	7.814727903
p値	0.442206279
判定	[ ]

■分散分析表

要因	自由度	偏差平方和	不偏分散	分散比	p値	判定
全体	23	15.73580026				
A	3	7.577120044	2.525706681	6.191458946	0.003773757	[**]
誤差	20	8.158680218	0.407934011			

■母平均の差の検定と推定

									信頼水準95%		信頼水準99%	
カテゴリー名1	カテゴリー名2	平均1	平均2	差	統計量	5%点	1%点	判定	下限値	上限値	下限値	上限値
コントロール	1g/kg	2.646946833	1.964132167	0.682814667	2.618686495	3.9583	5.0178	[ ]	−0.349300212	1.714929546	−0.625561667	1.991191
コントロール	1.5g/kg	2.646946833	1.179457	1.467489833	5.628021769	3.9583	5.0178	[**]	0.435374954	2.499604712	0.1591135	2.775866167
コントロール	2 g/kg	2.646946833	1.434381833	1.212565	4.650350593	3.9583	5.0178	[*]	0.180450121	2.244679879	−0.095811333	2.520941333
1g/kg	1.5g/kg	1.964132167	1.179457	0.784675167	3.009335274	3.9583	5.0178	[ ]	−0.247439712	1.816790046	−0.523701167	2.0930515
1g/kg	2 g/kg	1.964132167	1.434381833	0.529750333	2.031664098	3.9583	5.0178	[ ]	−0.502364546	1.561865212	−0.778626	1.838126667
1.5g/kg	2 g/kg	1.179457	1.434381833	−0.254924833	0.977671176	3.9583	5.0178	[ ]	−1.287039712	0.777190046	−1.563301167	1.0534515

◎ 母平均:テューキー(全群比較)

■基本統計量

カテゴリー名	n	合計	平均	分散(n-1)
コントロール	6	11.20326665	1.867211108	0.043812208
1g/kg	6	11.9329101	1.98881835	0.048310881
1.5g/kg	6	12.1162376	2.019372933	0.101024089
2 g/kg	6	11.78991465	1.964985775	0.092382999

■等分散性の検定(バートレット)

分布	カイ二乗分布
自由度	3
統計量	1.261579233
1%点	11.34486673
5%点	7.814727903
p値	0.738276045
判定	[ ]

■分散分析表

要因	自由度	偏差平方和	不偏分散	分散比	p値	判定
全体	23	1.505592333				
A	3	0.077941447	0.025980482	0.363961283	0.77972022	[ ]
誤差	20	1.427650886	0.071382544			

■母平均の差の検定と推定

									信頼水準95%		信頼水準99%	
カテゴリー名1	カテゴリー名2	平均1	平均2	差	統計量	5%点	1%点	判定	下限値	上限値	下限値	上限値
コントロール	1g/kg	1.867211108	1.98881835	−0.121607242	1.11490805	3.9583	5.0178	[ ]	−0.553354007	0.310139524	−0.668917683	0.4257032
コントロール	1.5g/kg	1.867211108	2.019372933	−0.152161825	1.395035701	3.9583	5.0178	[ ]	−0.583908591	0.279584941	−0.699472267	0.395148617
コントロール	2 g/kg	1.867211108	1.964985775	−0.097774667	0.896408482	3.9583	5.0178	[ ]	−0.529521432	0.333972099	−0.645085108	0.449535775
1g/kg	1.5g/kg	1.98881835	2.019372933	−0.030554583	0.280127651	3.9583	5.0178	[ ]	−0.462301349	0.401192182	−0.577865025	0.516755858
1g/kg	2 g/kg	1.98881835	1.964985775	0.023832575	0.218499568	3.9583	5.0178	[ ]	−0.407914191	0.455579341	−0.523477867	0.571143017
1.5g/kg	2 g/kg	2.019372933	1.964985775	0.054387158	0.498627218	3.9583	5.0178	[ ]	−0.377359607	0.486133924	−0.492923283	0.6016976

◎ 母平均:テューキー(全群比較)

■基本統計量

カテゴリー名	n	合計	平均	分散(n-1)
コントロール	6	1.97319956	0.328866593	0.001844998
1g/kg	6	2.14367492	0.357279153	0.005268906
1.5g/kg	6	1.92412332	0.32068722	0.006493382
2 g/kg	6	2.25990812	0.376651353	0.006498497

■等分散性の検定(バートレット)

分布	カイ二乗分布
自由度	3
統計量	2.040909395
1%点	11.34486673
5%点	7.814727903
p値	0.563959369
判定	[ ]

■分散分析表

要因	自由度	偏差平方和	不偏分散	分散比	p値	判定
全体	23	0.112534602				
A	3	0.012005692	0.004001897	0.796168481	0.510409232	[ ]
誤差	20	0.10052891	0.005026445			

■母平均の差の検定と推定

									信頼水準95%		信頼水準99%	
カテゴリー名1	カテゴリー名2	平均1	平均2	差	統計量	5%点	1%点	判定	下限値	上限値	下限値	上限値
コントロール	1g/kg	0.328866593	0.357279153	−0.02841256	0.981647359	3.9583	5.0178	[ ]	−0.142980623	0.086155503	−0.173646531	0.116821411
コントロール	1.5g/kg	0.328866593	0.32068722	0.008179373	0.282595452	3.9583	5.0178	[ ]	−0.106388689	0.122747436	−0.137054597	0.153413344
コントロール	2 g/kg	0.328866593	0.376651353	−0.04778476	1.650952376	3.9583	5.0178	[ ]	−0.162352823	0.066783303	−0.193018731	0.097449211
1g/kg	1.5g/kg	0.357279153	0.32068722	0.036591933	1.264242811	3.9583	5.0178	[ ]	−0.077976129	0.151159996	−0.108642037	0.181825904
1g/kg	2 g/kg	0.357279153	0.376651353	−0.0193722	0.669305017	3.9583	5.0178	[ ]	−0.133940263	0.095195863	−0.164606171	0.125861771
1.5g/kg	2 g/kg	0.32068722	0.376651353	−0.055964133	1.933547828	3.9583	5.0178	[ ]	−0.170532196	0.058603929	−0.201198104	0.089269837

Data of Table 2

Interscapular BAT

		Leptin	Adiponectin	UCP1	UCP2
		minus BTS	minus BTS	minus BTS	minus BTS
		0.0313	0.8645	2.8481	0.1001
		0.0147	0.5743	2.5253	0.0625
		0.0321	1.2058	3.6808	0.0764
		0.0290	1.0353	2.9548	0.0442
		0.0340	0.8293	2.2650	0.0567
		0.0194	0.6598	1.3430	0.0480
		0.0306	0.4931	0.7994	0.0928
		0.0328	0.4965	1.2482	0.0967
		0.0142	0.7526	2.6027	0.0679
	Average	0.0264	0.7679	2.2519	0.0717
	SD	0.0080	0.2436	0.9376	0.0210
		plus BTS	plus BTS	plus BTS	plus BTS
		0.1022	2.0849	3.0314	0.0511
		0.0819	0.9526	3.3404	0.0934
		0.2269	7.5685	3.5801	0.0529
		0.0638	1.0425	4.3169	0.1843
		0.1174	1.3472	3.0951	0.0994
		0.0508	1.5157	4.3469	0.0928
		0.1119	4.0558	3.5801	0.0583
		0.1908	1.3755	2.7511	0.0461
			2.9282	2.3295	0.0713
	Average	0.1182	2.5412	3.3746	0.0833
	SD	0.0612	2.1369	0.6704	0.0430
	t-test	0.0004	0.0250	0.0100	0.4781

Epididymal WAT					
		Leptin	Adiponectin	UCP1	UCP2
		minus BTS	minus BTS	minus BTS	minus BTS
		0.059839556	0.979420298	0.001975646	0.143587294
		0.078686769	1.328685814	0.002787514	0.166085727
		0.045341342	1.101905116	0.001640363	0.148650889
		0.02105	0.986232704	0.000621989	0.163799175
			0.835087919	0.00134768	0.133046273
			0.046070913	0.000860686	0.132127255
			0.089622203		0.210224104
			0.070316155		0.150725978
			0.066063628		
	Average	0.0512	0.6115	0.0015	0.1560
	SD	0.0243	0.5319	0.0008	0.0252
		plus BTS	plus BTS	plus BTS	plus BTS
		0.063486129	0.246558176	0.000598734	0.348685917
		0.03082747	0.840896415	0.00443636	0.116629124
		0.039693829	1.613283518	0.001314438	0.119908015
		0.030412	0.939522749	0.002076004	0.26425451
		0.049774159	1.148698355	0.002473187	0.210224104
		0.007115	1.049716684	0.000944429	0.156041319
		0.0669375	0.864537231		0.122427537
		0.0234145	1.484523571		0.040107059
			0.267943366		0.220675749
	Average	0.0390	0.9395	0.0020	0.1777
	SD	0.0203	0.4682	0.0014	0.0930
	t-test	0.3756	0.1839	0.5207	0.5347
